# Overview of Engineering Carbon Nanomaterials Such As Carbon Nanotubes (CNTs), Carbon Nanofibers (CNFs), Graphene and Nanodiamonds and Other Carbon Allotropes inside Porous Anodic Alumina (PAA) Templates

**DOI:** 10.3390/nano13020260

**Published:** 2023-01-07

**Authors:** Leandro Nicolás Sacco, Sten Vollebregt

**Affiliations:** Department of Microelectronics, Faculty of Electrical Engineering, Mathematics and Computer Science, Delft University of Technology, Feldmannweg 17, 2628 CT Delft, The Netherlands

**Keywords:** porous anodic alumina, anodization, carbon nanotubes, carbon nanofibers, graphene, nanodiamonds

## Abstract

The fabrication and design of carbon-based hierarchical structures with tailored nano-architectures have attracted the enormous attention of the materials science community due to their exceptional chemical and physical properties. The collective control of nano-objects, in terms of their dimensionality, orientation and size, is of paramount importance to expand the implementation of carbon nanomaterials across a large variety of applications. In this context, porous anodic alumina (PAA) has become an attractive template where the pore morphologies can be straightforwardly modulated. The synthesis of diverse carbon nanomaterials can be performed using PAA templates, such as carbon nanotubes (CNTs), carbon nanofibers (CNFs), and nanodiamonds, or can act as support for other carbon allotropes such as graphene and other carbon nanoforms. However, the successful growth of carbon nanomaterials within ordered PAA templates typically requires a series of stages involving the template fabrication, nanostructure growth and finally an etching or electrode metallization steps, which all encounter different challenges towards a nanodevice fabrication. The present review article describes the advantages and challenges associated with the fabrication of carbon materials in PAA based materials and aims to give a renewed momentum to this topic within the materials science community by providing an exhaustive overview of the current synthesis approaches and the most relevant applications based on PAA/Carbon nanostructures materials. Finally, the perspective and opportunities in the field are presented.

## 1. Introduction

During recent decades, carbon nanomaterials synthesis and applications arouse as a very attractive and growing field of science, due to their exceptional optical [1], electrical [2,3], mechanical [4,5], thermal [6] and sensing [7,8,9,10] properties. In particular, for a wide range of applications, such as nanoelectronics devices [11], filtration membranes [12,13], photodetectors [14], and charge storage [15] the fabrication of a membrane with uniform pores is critical for achieving better performance than membranes composed of randomly distributed bundles of carbon (or other) nanostructures. Compared to the relative expensive lithography-based approaches [16], template-based synthesis approaches provide a relatively simple route to prepare scalable 0-D and 1-D nanostructure arrays.

Various templates have been implemented, such as mesoporous carbon [17], polymer matrixes [18], zeolites [19], nanochannel arrays in porous silicon and glass [20,21], and anodized metal oxide template [22,23,24,25]. Porous anodic alumina (PAA) templates have been demonstrated as an outstanding host matrix for the production of carbon nanostructures membranes with controllable dimensions [26], as a result of their thermal and chemical stability, mechanical compliance, and relatively straightforward fabrication method [27]. In the literature, these porous templates are also called anodic aluminium oxide (AAO) or nanoporous anodic alumina (NAA). This review paper will refer to the porous ordered alumina oxide as PAA. A key benefit of PAA templates in comparison with other template-based approaches [28], relies on the fact that the geometrical features of a highly ordered close-packed array can be precisely engineered by adjusting the parameters involved in the anodization process [29,30].

**Figure 1:**
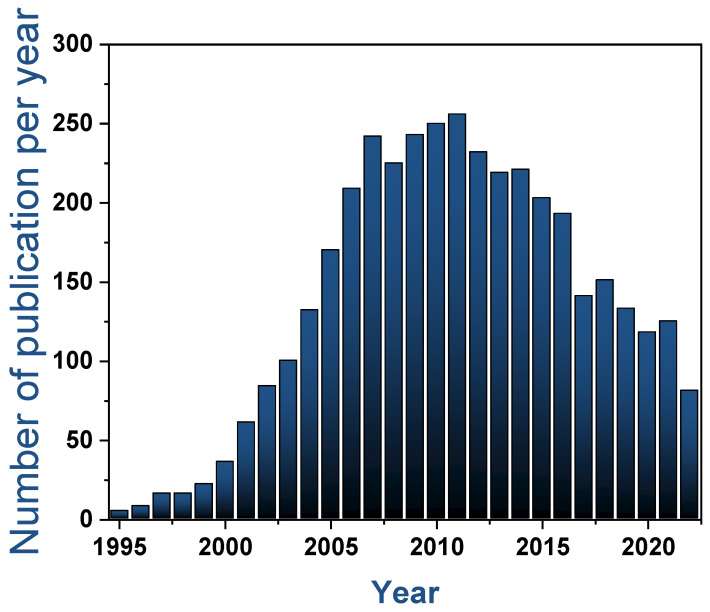
The number of articles published on porous anodic alumina. Source: Web of science. The search was carried out in November 2022.

However, as Figure 1 reveals, during the last eight years the number of articles published of porous anodic alumina has suffered a decline followed by a more stable period in recent years. This trend can be correlated with the boom of 2D materials that seldom requires the use of nanostructured templates. Nonetheless, there are still many applications that require the use of PAA templates [31,32,33,34,35] and novel hybrid materials such as metals sulphides [36] and metal alloys nanowires [37] that are suitable for nanostructured templates.

Very interesting review articles were published recently that provide an extensive description of the signs of progress made on the PAA fabrication [38,39], characterization [40], formation mechanisms [41,42] and applications [43,44]. All these works agree that the porous materials based on alumina templates are a very attractive platform to develop a wide range of applications but are still fundamental to get a better understanding of their growth mechanisms to gain more advanced control over their structure and the interaction with other materials. Consequently, much effort must be devoted to obtaining a better understanding of the anodization process and the influence of the PAA features on the synthesis of nanomaterials within the nanopores. In this direction, many works performing in-situ time-resolved X-ray scattering observations have been carried out to shed light on the development of the self-ordering pores process [45,46,47,48]. Besides, theoretical models are required to precisely establish a theoretical linking between the long-range order of the PAA templates during their formation process and their physicochemical properties. In this perspective, an interesting work performed by S. Heinschke and J. Schneider [49] provides some guidelines that serve as a starting point for further theoretical investigations in this direction. Regarding the synthesis of carbon-based nanomaterials within PAA templates, there are still some challenges that slowed down their production on an industrial scale. Despite the fact the current anodized technology has been implemented to continuously develop novel PAA-based devices exploited for synthesizing a variety of applications, this technology is still significantly limited by the difficulty in engineering the required morphology in the nanoscale (e.g., pore diameters below 15 nm, thin PAA layers, removal of oxide dielectric layer).

**Figure 2:**
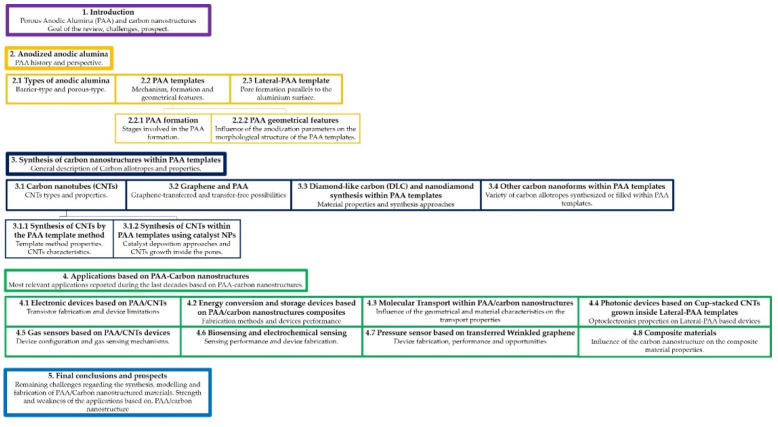
Structure diagram of the present review article.

With respect to recently published literature, the present review article aims to focus on the fabrication/synthesis of PAA templates with carbon nanostructures that so far was not been exclusively reported in spite of the massive amounts of work devoted to the fabrication of these hybrid materials. Besides, this review’s intention is to bring renewed attention to the fabrication of carbon-based materials synthesized within PAA templates. Reaching out to both, newcomers and experienced researchers in the area of PAA and carbon materials. Summarizing and identifying the opportunities and limitations of the carbon nanoforms that can be synthesized inside PAA templates. Then, this review provides a complete description of the state-of-art pointing out the main challenges related to PAA fabrication, the further growth of carbon materials and subsequent characterization. As a result, the review will begin with a general description of the PAA covering the alumina formation and the main geometrical features, which can determine the synthesis of carbon nanomaterials on the template (Section 2). Next, it discusses the different carbon-based materials synthesized and transferred onto PAA templates, such as CNT, graphene, CNFs, diamond-like carbon (DLC) and nanodiamonds. For each material, the main advantages and drawbacks are analyzed and their specific strategies to engineering PAA/Carbon-based material are discussed (Section 3). Furthermore, the main applications based on PAA/Carbon-based devices are summarized by analyzing their performance (Section 4). Finally, the present review paper concludes by discussing the challenges and opportunities of the PAA carbon-based materials and the perspective of the field (Section 5). To guide the reader through the presented review article, Figure 2 shows a structure diagram indicating the sections and subsections of the article.

## 2. Anodized Anodic Alumina

During the anodization process aluminium is used as an anode in an electrochemical cell, the electrochemical cell is immersed in acidic solutions and an electric field is applied between a cathode (classically graphite or Pt). Depending on the anodization conditions a self-organized porous oxide film is formed [50]. Further details about the types of anodization processes and the mechanism involved are later discussed. The improvements in the electron microscopy field allow a detailed characterization of the geometrical features of the PAA [51]. The research article performed in 1970 by O’Sullivan and Wood [52] provided new insights into the porous anodic formation and morphology, making a crucial step towards a comprehensive understanding of the PAA formation. Until the 1990’s most of the efforts were concentrated to obtain a detailed description of the PAA and an explanation of the mechanisms involved in the PAA fabrication. An essential innovation was achieved by Masuda and Fukuda [53] when they proposed the two-step anodization process (see Section 2.2.1). This straightforward technique has significantly improved the PAA organization leading to a template with a highly ordered close-packed array. Here the geometrical features of the template can be precisely controlled by adjusting the parameters involved in the fabrication process [54,55,56]. Since the two-step anodization process was adopted, a lot of progress has been performed in terms of template tailoring and the mechanism involved in the self-organized pore array formation.

In summary, the study of the PAA has opened new frontiers for template fabrication since the numerous advantages that provide this kind of porous structure, such as the pore ordered distribution, the wide range of possible pore aspect-ratios, the easy control of the structural parameters of the template, and the low costs associated to the implementation of the anodization process. For further details about the history of science and technology that deals with the development of the aluminum anodizing industry, the reader is referred to the book by Runge [56] which provides an exhaustive description of the aluminum anodization research carried out from the nineteenth century up to the most recent advances.

### 2.1. Types of Anodic Alumina

**Figure 3:**
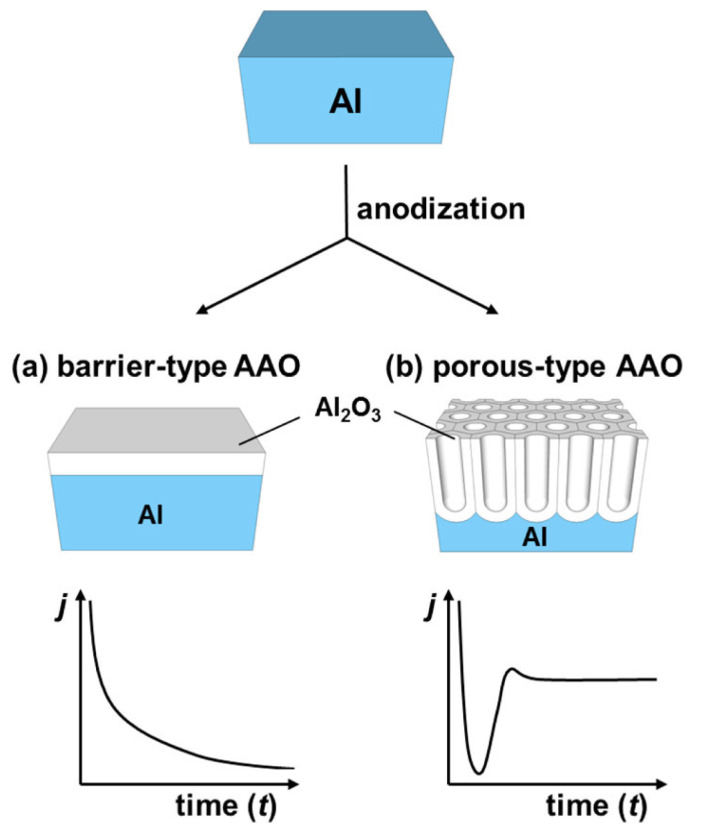
Two sorts of anodic alumina films (**a**) barrier-type and (**b**) porous-type with their respective anodization current curves as time evolves under potentiostatic conditions. The two types of anodic alumina are reprinted with permission from Ref. [30]. Copyright 2014 American Chemical Society.

Depending mainly on the acidic level of the aqueous electrolyte, two different kinds of anodic aluminum oxide can be obtained: the barrier-type anodic alumina or the porous-type anodic alumina, as schematized in Figure 3. For basic or neutral pH solutions, barrier-type anodic alumina is formed, as shown in Figure 3a. In this case, the native barrier layer is growing until it reaches a critical thickness from which the process cannot evolve anymore because the strength of the electric field is not strong enough to transport the ions through this insulating layer. Nowadays, thin anodic oxide films in the nanometer scale are important for the insulation and encapsulation of components in advanced micro/nanoelectronic devices [57,58]. The present review mentions the barrier-type anodic alumina for sake of completeness because the main objective is to explore the porous nature of the anodic alumina oxide as shown in Figure 3b. Nevertheless, there are interesting research articles focused on the barrier-type anodic alumina that cover the dynamics involved during the dielectric formation and their applications [59,60,61].

### 2.2. PAA Templates

#### 2.2.1. Porous Anodic Alumina Formation

Highly ordered PAA films are fabricated by the anodization process of an aluminium substrate in an acidic solution under either a potentiostatic or galvanostatic condition. The PAA formation is characterized by three stages: pore nucleation, pore growth and self-organization. There is a vast literature covering the PAA formation at the different stages [30,62,63,64], describing the pore nucleation [65,66,67], the subsequent pore growth [52,68,69,70,71] and finally the self-organization of a porous structure in a long-range ordered with a closed packaged honeycomb-like pattern [49,72,73,74].

**Figure 4:**
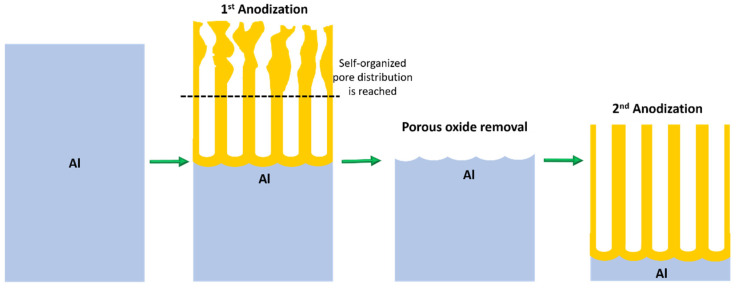
Steps involved in the two-step anodization process.

The PAA template implementation as a nanostructure was revolutionized after the methodology introduced by Masuda and Fukuda [48] named the two-step anodization process. The presented method is based on aluminum substrate pre-texturing obtained by the removal of a PAA sacrificial layer. In a first attempt, a PAA is formed using specific potentials for certain electrolytes and sufficiently long anodization times that guarantee a self-organized pore formation. Under these conditions, the distribution of the pores at the bottom has a higher degree of order than the pore distribution at the top surface of the alumina. After the first PAA layer is removed with an etchant solution that selectively removes the alumina, the aluminum substrate remains with nanoimprints created by the removed pores at the bottom with a honeycomb-like pattern distribution. Finally, a second anodization process is carried out, but in this case, the electric field lines are focused on the nanoimprints, therefore, the pore formation begins following a hexagonal array. Figure 4 summarizes the two-step anodization process.

Even after the introduction of the two-step anodization process, the underlying principle of self-ordering growth regimes remains a pending challenge in the field of PAA templates. Although the research efforts devoted during the last decades, the self-organization phenomenon still does not have a consistent theoretical framework that precisely model and predict the evolution in the formation of a self-organized hexagonal closed packing structure. One of the major difficulties is the intrinsic multifarious nature of the anodized aluminium process. Interestingly, Pashchanka [42] points out that interdisciplinary approaches need to be considered, entailing materials science, electrochemistry, hydrodynamics, and elements of chaos and self-organization theory. 

The attempts to describe the pore growth in a steady-state were based on the interaction of two complex processes like field-assisted alumina dissolution under the application of an electric field in an acidic environment generally obtained by an electrochemical approach, and the stress inside the alumina formed at the aluminium surface. However, this field-assisted hypothesis has been disregarded [70] by a model that attributes the self-organization due to mechanical constraints triggered by the different expansion volumes of Al_2_O_3_ (alumina) with respect to the Al.

Following this idea, Nielsch et al. [64] proposed the 10% rule, indicating that independently of the specific anodization conditions, achieving the self-ordering of the porous alumina requires a porosity of 10%. This relation between the pore surface over the total area of the alumina film is associated with a volume expansion of alumina to aluminium of about 1.2. This discovery provided a connection between an “ideal” cell pore parameter and a set of anodization conditions to be adjusted to obtain the best pore formation conditions.

Then, Roslyakov et al. [74] developed a very interesting model that takes into consideration electrode reaction kinetics as a key factor for the evolution of a self-organized honeycomb-like porous structure. The main conclusion relies on the fact that only an ordered porous distribution is obtained when the migration of the ions takes place at the barrier layer which separates the aluminium and the electrolyte at the pore bases, or when the diffusion of ions occurs through the PAA pores. In contrast, when a mixed control of both phenomena occurs during the anodic oxidation process disordered porous structures are formed.

Recently, Heinschke and Scheineder [49], for the first time, tackled the PAA growth evolution into a framework of irreversible thermodynamics with a special focus on the abstract concept of entropy production. The main conclusions are that the pore growth is limited by parameters that depend on migration within the electrolyte/bulk interphase and that convection and diffusion terms do not contribute to the self-organization of the PAA structure. Their work forms the basis for future theoretical models using a new approach based on the investigation of entropy production and thermodynamic notions. This kind of approach is very useful to theoretically link the order of the cell arrangement and the PAA forming processes. Several interesting recent reviews articles focus on the anodic alumina templates. Ruiz-Clavijo et al. [43] and Domagalski et al. [44] provide a detailed updated state-of-the-art review where they also discussed the pioneering works up to the most recent advances of the studies devoted to the mechanism involved in the pore formation and the ordering of the pores in a close-packed hexagonal arrangement.

To further obtain experimental insights into the dynamic processes of PAA formation, nowadays many studies are focused on the In situ structural studies of PAA films using grazing-incidence transmission small-angle X-ray scattering [46,75], optical emission spectrometry [76] and electrochemical measurements [77]. Linpé et al. [47] investigated the in situ electrodeposition of Sn pillars within PAA templates with grazing transmission small-angle X-ray scattering, X-ray fluorescence, and X-ray absorption near edge structure spectroscopy. During the last years, TEM observation in situ and *operando* conditions of a wide diversity of carbon nanomaterials were successfully carried out [38,78,79,80,81]. The *in-situ* observation and characterization of carbon nanostructures into PAA is an outstanding challenge across the material science community that must be considered to introduce new insights into the influences of the PAA structure on the synthesized structure.

Despite these advancements, there are remaining challenges related to a precise description of the porous hexagonal ordered anodic alumina. There is a massive amount of literature to precisely control the geometrical features of the PAA such as the pore cell size, pore diameter, interpore distance, oxide pore thickness at the bottom of the pores, porosity and circularity of the pores, making the PAA an outstanding platform for the synthesis of nanostructures. An exhaustive overview of the different possible architectures is given in recent reviews focused on the developments of nano porous alumina templates [38,40,43,82].

#### 2.2.2. PAA Geometrical Features

This section focuses on the main geometrical features of the PAA templates and the influence of the anodization parameters on the film morphology. Then, good control of the parameters involved during the anodization is critical to fully exploit the use of PAA as a template. It is important to highlight that porous morphology can serve to collectively confine carbon nanostructures but also can determine their size or constrain the growth evolution of nano-objects [83].

**Figure 5:**
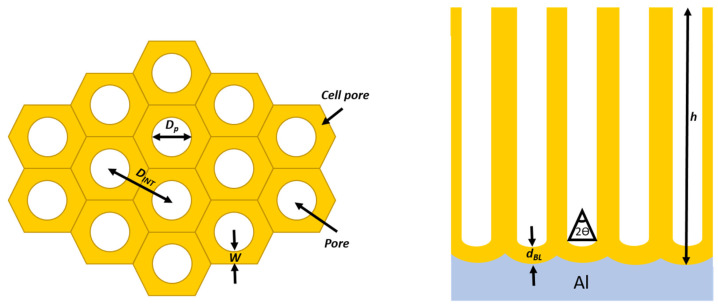
Main geometrical features of a PAA template.

Figure 5 shows a detailed schematic PAA template pointing out the cell pore and their respective six structural parameters that characterized the PAA. Herein, we briefly describe each structural parameter and how they are affected by the anodization conditions.

The pore diameter ($d_{p}$), remains constant through the whole pore height under a steady-state anodization regime. The major anodization parameter that affects the $d_{p\;}$ is the anodization potential. Many studies report a linear relationship between the applied anodization potential (U) and $d_{p}$ scaled with a constant $\lambda_{P}$ [51,84,85] as expressed by Equation (1):

(1) $$d_{p}=\lambda_{P}\cdot U$$

In principle, the pore diameter is not sensitive to the anodizing electrolyte. However, it is important to highlight that the electrolyte type determines the best pore ordering degree of the self-organized PAA honeycomb-like structure. The most common electrolytes are 0.3 M oxalic acid, 0.2 M sulphuric acid and 0.3 M phosphoric acid. In these three cases, the anodization processes performed under mild conditions generally involve potentials of 25 V, 40 V and 195 V, respectively for each kind of acid solution [54]. 

In hard-anodization conditions, the used potentials are to a certain limit higher, depending on the electrolyte. This sort of anodization is more advantageous in terms of processing times since the oxide growth rates are 2500–3500% faster than PAA obtained via mild anodization. Nevertheless, hard anodization is associated with abundant gas bubbles production at the bottom of the pores. The bubbles can dramatically hinder ion diffusion, which will lead to the irregular arrangement of the pore cell and disrupts the self-ordering process. In many cases increasing the potential above 160 V can lead to sample burning [86]. To overcome this drawback, the addition of alcohols (typically ethanol or methanol) to the electrolyte bath arises as a solution. The alcohol addition to the chemical cell enables a considerable decrease in the freezing point of the diluted (water-based) electrolyte. Therefore, anodization temperatures below 0 °C are possible to decrease the electrolyte temperature and more efficiently dissipate Joule heating at the pore bottoms [87]. In any case, for both kinds of anodization conditions, it was verified that the pore diameter follows a linear dependence with the anodization potential, where different $\lambda_{P}$ were reported ranging from 0.47 nm/V [88] up to 1.38 nm/V [89].

The interpore distance ($d_{INT}$) is defined as the distance between two consecutive pores and also linearly depends on the applied potential during the anodization process as is expressed in Equation (2):

(2) $$d_{INT}=\lambda_{INT}\cdot U.$$

Interestingly, Lee et al. [90] used the so-called hard anodization approach to fabricate pore cell structure with long inter-pore distances ($d_{INT}$ = 200–300 nm) that cannot be achieved by mild anodization. Furthermore, this work points out that processes carried out under mild conditions $\lambda_{INT}^{Mild}$~2.5 nm/V are rather independent of the applied electrolyte [30], while for hard anodization the electrolyte can affect the inter-pore distance [91]. Also, the linear dependence is scaled differently, where the proportional constant, in this case, is $\lambda_{INT}^{Hard}$~2.0 nm/V.

The oxide barrier layer is present under the pore as shown in Figure 5. The thickness ($d_{BL}$) of this dielectric layer is defined as the distance between the Al/Al_2_O_3_ interphase and the Al_2_O_3_/electrolyte interphase during the anodization process. The barrier layer has the same stoichiometry as the native oxide film of aluminium exposed to the atmosphere. This compact barrier layer at the pore bottoms determines any further applications based on electrodeposition or electrical contacts through the alumina layer since all transport current needs to cross through the barrier. Thus, precise control of such structural parameters is crucial for many applications [14,92,93,94]. Many methods have been developed to thin the barrier layer [95,96] or to completely dissolve it [97,98,99]. It has been proven that the thickness of the dielectric layer at the pore’s bottom is proportional to the applied potential [52], as shown in Equation (3). However, it was not reported to be sensitive to the anodization temperature [100,101] as was originally postulated by the pioneering work from O’Sullivan and Wood [52].

(3) $$d_{BL}=\lambda_{BL}\cdot U$$

The proportional constant also depends on the type of anodization. For instance, it was widely reported that for mild anodization $\lambda_{BL}^{Mild}$~1.3–1.4 nm/V [99,102,103], while for hard anodization $\lambda_{BL}^{Hard}$~1.0 nm/V [90]. The different scaling is attributed to higher current densities involved in the hard anodization process that are related to the anodization ratio (AR) that expresses the dependence between the barrier layer thickness and the applied potential, defined as $AR\;=\;\frac{d_{BL}}{U}$ [104]. The density of current across the barrier layer is proportional to the density of current $J\;\alpha \;exp\left(\beta U/d_{BL}\right),$ where *β* is a material-dependent constant. Therefore, for a given anodization condition, the oxide dielectric layer thickness is inversely proportional to the logarithm of current density. 

The pore wall thickness (W) has been proven to be thinner than the oxide barrier layer at the pore bottom’s scaled with a factor α varying from 0.7–0.91 [51,52,88,105]. To get back to the point regarding the inter-pore distance scale proportionality, the underlying dependence with the anodization type can be expressed in terms of the wall thickness and with the oxide barrier layer present at the pore’s bottom as is expressed in Equation (4):

(4) $$d_{INT}=\lambda_{INT}\cdot U=2\cdot W+D_{P}=\alpha \cdot d_{BL}+D_{P}=\left(\alpha \cdot \lambda_{BL}+\lambda_{P}\right)U\;$$

**Figure 6:**
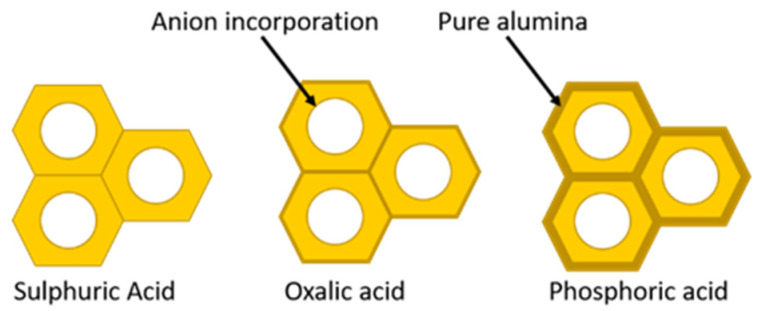
Schematic representation of the duplex pore wall structure characterized by the inner and outer pore cell structure depending on the anodized electrolyte: Sulfuric acid, Oxalic acid and Phosphoric acid. The thickness of the inner layer varies depending on the kind of electrolyte used during the anodization process.

An important feature of the pore wall is its duplex structure, as schematized in Figure 6. Schematic representation of the duplex pore wall structure characterized by the inner and outer pore cell structure depending on the anodized electrolyte: Sulfuric acid, Oxalic acid and Phosphoric acid. The thickness of the inner layer varies depending on the kind of electrolyte used during the anodization process, where the cell structure contains two regions separate by a pure compact alumina wall and anion incorporated layer. It was reported that the thickness of the inner layer varies depending on the electrolyte implemented in the succeeding order [105]: H_2_SO_4_ < C_2_H_2_O_4_ <H_3_PO_4_. Besides the influences of the electrolyte, Han et al. [106] verified that the anodization duration impacts the anion distribution through the pore length. For two-step anodization processes longer than 12 h performed by using 0.3 M H_2_C_2_O_4_ (7 °C) as an electrolyte at 40 V, a lower amount of anion impurity content is incorporated into the barrier oxide layer of PAA film, in contrast to those PAA fabricated with a shorter anodization time, ranging from 0.5–8 h, which present a high amount of anions contents. This is a remarkable finding since the anion density within the pore walls directly affects the pore widening etching, a widespread method to enlarge the pore cell structure and reduce the compact oxide layer at the bottom of the pore.

The pore length (h) is the distance between the alumina’s top surface and the surface at the bottom of the pore. This length depends on the chemical reaction rate at the bottom of the pores, as previously described, the anodization type (hard or mild), the electrolyte concentration, the electrolyte temperature, and of course the anodization duration. 

Finally, the central angle (θ) is determined by the radius of curvature of the pore base. Basically, for 90° the central angle the radius curvature of the bottom of the pores is equivalent to a half-sphere, and for lower angles, the pore curvature corresponds to a smaller fraction of a half-sphere. An angle equal to 90° implies that the pore wall and the dielectric barrier thicknesses are equivalent, but, as previously mentioned, the pore wall thickness is thinner than the oxide barrier layer. This can be attributed to the fact that it is always required a vertical electric-field component at the pore junction during the alumina formation at the bottom of the pores. This geometrical feature was studied in depth in the work performed by Li et al. [107].

The previously introduced geometrical parameters are inherent to the pore cell structure. There are also key parameters that are related to the collective organization. For instance, the porosity P, expresses the ratio between the area occupied by pores and the alumina surface. For an ideal hexagonally arranged cell it can be expressed in terms of the inter-pore distance and pore diameter (Equation (5)) [108]. The pore density n described the number of pores per unit of area, once again, considering a perfect hexagonal, the density of pores occupying the surface area of 1 cm^2^ is expressed by Equation (6) in terms of the inter-pore distance.

(5) $$P=\frac{\pi }{2\sqrt{3}}\cdot {\left(\frac{D_{C}}{D_{P}}\right)}^{2}$$

(6) $$n=\frac{2\times 10^{14}}{\sqrt{3}\cdot D_{C}{}^{2}}$$

Finally, there are parameters to quantify how the formed PAA structure differs from an ideal hexagonal cell structure. For instance, the circularity of the pores C parametrized how the pores differ from an ideal circular pore as expressed by Equation (7), where a pore occupied a surface area (*S*) to squared perimeter ratio (*L*) [109]. The circularity value of 1.0 indicates that the pore’s shape is a perfect circle, while values close to 0.0 indicate the presence of a deformed irregular shape.

(7) $$C=4\pi \cdot \left(\frac{S}{L^{2}}\right)$$

Different strategies based on image analysis and further data treatment have been developed to estimate the ordering of the porous hexagonal structure. Regularity ratios are obtained from top-view images of the nanoporous alumina inspected by scanning electronic microscopy (SEM). Fast Fourier transform (FFT) based quantitative arrangement analysis is widely implemented to measure periodic structures [110,111] where the maximum of the intensity profile from the FFT image is divided by the width of the peak at half of its height. Similarly, self-correlation functions have been applied to SEM images [112] where a mathematical image in the real space is obtained implying that from the self-correlation image geometrical characteristics, such as inter-pore distances and particularly pore diameters can be directly extracted. Delaunay triangulations have also been used to identify a not six-fold coordinated by neighbouring pores and further associated as a defect in the hexagonal array [113]. Interestingly, Toccafondi et al. [114] have developed an efficient method based on regularity ratio calculation extracted from radial profiles of FFT of the images and further Minkowski calculations. This methodology allows calculating the ordering of PAA film differing from a hexagonal pattern that can be obtained when a single anodization step is obtained, or the anodized aluminium thickness is too short.

### 2.3. Lateral-PAA Template

**Figure 7:**
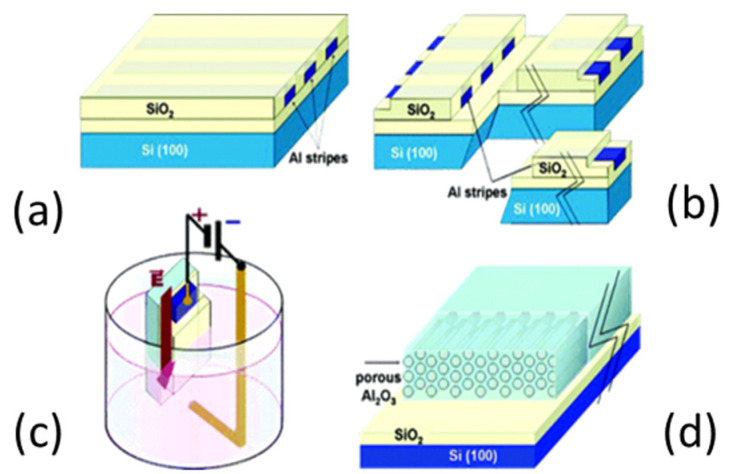
Sample processing and experimental setup for the fabrication of lateral-PAA templates. (**a**) A thin film of aluminium is first deposited on an insulating substrate (e.g., an oxidized Si wafer) and etched into parallel stripes. The Al stripes are subsequently capped with an insulating layer (SiO_2_, SiN*_x_*, resist). (**b**) The capping insulating layer is also patterned (and etched) in large stripes, leaving the Al locally exposed. Al is then locally etched using the capping layer as a mask. The mask is also removed at the periphery to provide an electrical contact area to the Al thin film. Finally, the Si substrate is cleaved into several individual stripes. (**c**) Individual Al stripes (connected at the periphery) are partially immersed into the electrochemical bath for anodic oxidation. As a result of the engineered structure of (**b**), the anodic oxidation current is forced to flow parallel to the surface of the substrate. Hence the porous structure is also forced to develop parallel to the surface of the substrate as schematically shown in (**d**). The lateral-PAA templates sketch are reprinted with permission from Ref. [115]. Copyright 2005 American Chemical Society.

Multiple applications require a collectively ordered array in the direction parallel to the substrate. Typically, PAA films are anodized using aluminium foils or aluminium evaporated onto rigid substrates. In these cases, the pore formation occurs in the exposed surface (i.e., perpendicular to the substrate surface). To expand the application field of PAA templates, lateral-PAA templates have also been developed by Cojocaru et al. [115], where the pores evolve during the anodization parallel to the surface of the substrate, instead of perpendicular as are conventionally performed. This approach entails an encapsulation step of the aluminium in the horizontal orientation, to fully constrain the pore formation in the lateral direction. In this case, the pore growth dynamic is modified by the stress imposed by this insulating mask. Figure 7 sketches the process flow for fabrication of the structures for lateral-PAA templates.

**Figure 8:**
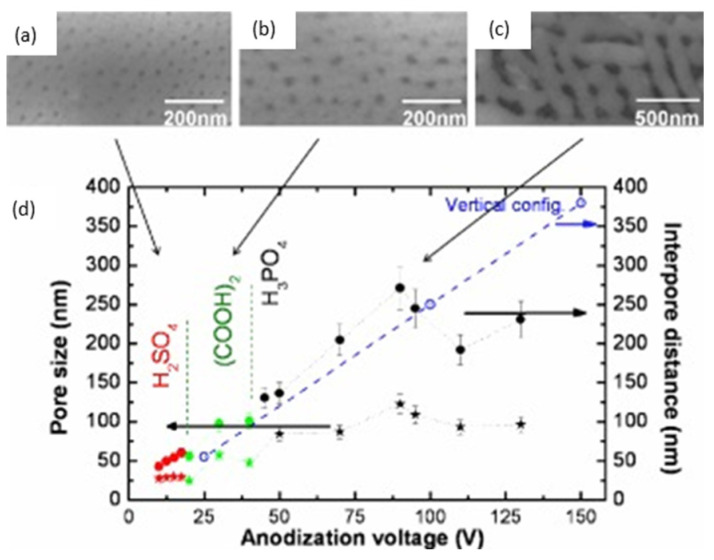
Representative SEM images of samples were obtained with the three most used types of electrolytes: (**a**) 0.3 M Sulfuric acid (20 V), (**b**) 0.3 M Oxalic acid (40 V), (**c**) 0.4 M Phosphoric acid (90 V). (**d**) Pore size (left axis) and interpore distance (right axis) of the horizontal porous alumina membranes as a function of the anodization potential. The results are compared with the conventional vertical configuration results (blue dashed line). Reprinted and modified with permission from Ref. [117]. Copyright © 2023 WILEY-VCH Verlag GmbH & Co. KGaA, Weinheim, Germany.

Regarding the anodic alumina formation in this horizontal geometry, very interesting work was performed by Oh and Thompson [116], which revealed an abnormal growth behaviour of PAA in this kind of confined configuration that leads to periodic dendritic pore structures and reduced growth kinetics instead of a smooth pore growth that takes place in the vertical devices. The authors attributed this pore formation due to the suppressed volume expansion and plastic deformation of the oxide in confined structures during anodization. A comparison between the most common anodization conditions in lateral-type and the conventional vertical-PAA templates has been performed by Xiang et al. [117]. Figure 8 shows representative SEM images of lateral-PAA obtained with three different electrolytes (Sulfuric acid, Oxalic acid, and Phosphoric Acid) with a plot depicting the relation of the pore diameter and interpore distance as a function of the anodization potential for each electrolyte. The results are compared with the conventional vertical configuration results (blue dashed line).

## 3. Synthesis of Carbon Nanostructures within PAA Templates

**Figure 9:**
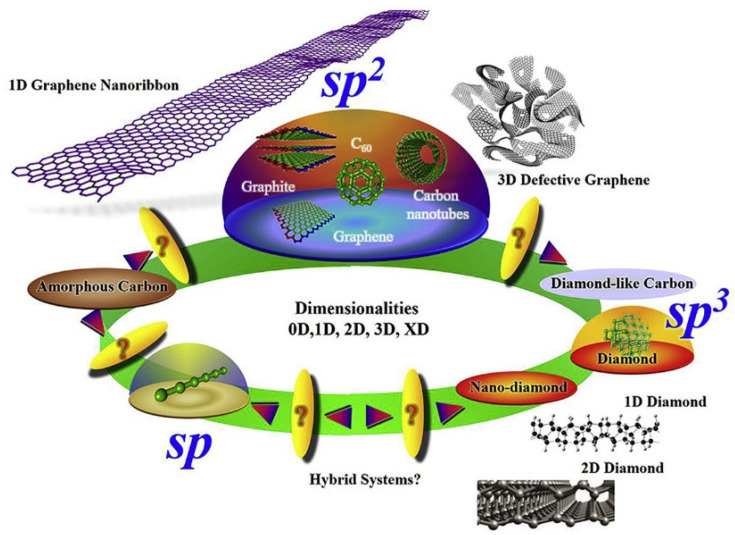
Carbon allotropes with different dimensionalities and hybridizations. Reprinted with permission from Ref. [118]. Copyright © 2023 Elsevier Ltd. All rights reserved.

Many carbon allotropes exist due to the multiple bond hybridization possibilities (sp, sp^2^, sp^3^) among carbon atoms. This remarkable flexibility of carbon elements to catenate leads to structures with diverse properties that are implemented in a wide range of applications [118]. The outstanding carbon allotropes properties rely on the fact that there exist structures ranging from zero dimensionality up to three dimensions, with different associated physical/chemical properties [119]. Figure 9 depicts a large family of carbon allotropes with different dimensionalities and hybridizations. The most representative case is the difference between graphite, which is characterized by its black opaque colour, softness and excellent electrical conductivity, and diamond, which is a transparent material, well-known as one of the hardest materials [120] and electrically insulating. The reader can refer to many recent review papers to gain new insights into the fascinating field of carbon allotropes, which provides an exhaustive description of the variety of synthesized carbon nanomaterials [121], and the current challenges for the design [122] and further synthesis of new carbon allotropes [118].

In the previous section, a detailed description of the geometrical features of the PAA and the influence of the parameters involved with the anodization process on their morphology was given. As mentioned, PAA templates offer an adaptable platform where carbon nanostructures can be collectively organized, confined, and can be easily tuned within the nano-sized channels. Here, we revisit the most relevant articles on nano-carbon allotropes growth within PAA templates. Furthermore, a brief introduction of the carbon nanostructures implemented in the PAA field points out their main properties and applications.

### 3.1. Carbon Nanotubes (CNTs)

From a structural point of view, a single-walled carbon nanotube (SWCNT) can be considered as a rolled-up cylindrical graphene sheet having a sp^2^ hybridization. Depending on the number of concentric tubes that form the structure, the tubes can be classified as single, double, or multi-walled carbon nanotubes (MWCNTs). The SWCNT diameter ìs typically in the range of a few nanometers, on the other hand, MWCNTs have an external diameter ranging from 2–100 nm depending on the number of the external concentric tubes [123]. The length can range from a few nanometers up to a few centimeters [124], then, aspect ratios of the order of 1,000,000 can be achieved. The astonishing mechanical, electrical, thermal, and chemical stability properties [125] make CNTs suitable to be deployed in a wide range of applications [126,127,128,129,130,131]. It is worth mentioning that the various CNTs properties are determined by how the carbon sheet is arranged, An interesting overview about the influence of the chiral vector on the properties of CNTs are summarized by Janas [132]. Therefore, one of the current challenges intrinsic to the SWCNTs based devices development depends on a precisely sorting and/or chirality control.

There are still drawbacks regarding the CNTs applications that requires suspensions because the intertube forces are ruled by Van der Waals interaction that leads to the formation of bundles or aggregates. CNTs sidewalls modification is required to overcome this issue [133]. In addition, one of the major challenges regarding the CNTs synthesis remains their collective organization. During the last decades, many works focused on the synthesis of vertically aligned carbon nanotubes (VA-CNTs) [134,135,136]. However, only some applications require the bulky properties of the vertically aligned assembly of tubes. Many applications rather need a surface modification step to synergically benefit from the bulky platform of the VA-CNTs with a specific functionalization [134,137]. Plasma-enhanced CVD (PECVD) is potentially the most suitable to synthesize VA-CNT arrays as the tube alignment is guaranteed by the electric field induced by the plasma, and therefore are no limitations regarding the intertube distance or equivalently the catalyst distribution as it is the case for pure thermal CVD processes. However, it’s worth mentioning that the radicals and ion bombardment involved during the plasma process tend to generate a large number of defects in the crystal tube structure [138,139]. Consequently, the PAA templates offer an attractive approach to collectively organize CNTs in a wide range of densities and are compatible with further functionalization [140,141]. Then, the PAA-CNTs materials can offer a versatile platform where the control of the CNTs morphology and position can be engineered. 

CNTs growth inside porous templates was reported for the first time by Kiotany et al. [142]. The implemented synthesis approach consists of the deposition of an amorphous carbon structure on the internal pore walls by the high-temperature pyrolysis of a carbon feedstock. Alternatively, there is a second approach, where a metal catalyst is present in the PAA template and the ensemble of PAA/catalyst is used to grow CNTs by chemical vapour deposition (CVD) [143]. Both approaches have been widely implemented mainly targeting a specific application (for energy storage-related applications the template approach was mainly adopted, and for electronic applications was generally used a catalyst-assisted method) since there are strengths and weaknesses inherent to both fabrication methods.

#### 3.1.1. Synthesis of CNTs by the PAA Template Method

**Figure 10:**
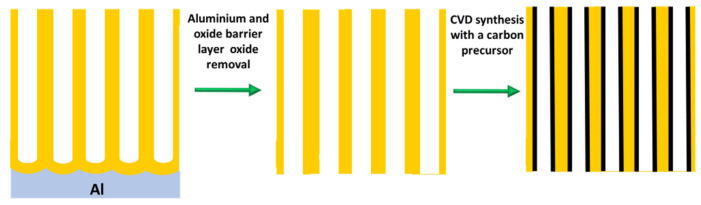
Methodology for the catalyst-free synthesis of CNTs within PAA templates. The black lines represent the CNTs growth on the PAA walls.

In catalyst-free CVD processes within PAA templates, carbon precursors are deposited on the pore walls by thermal decomposition of hydrocarbons. The main advantage of this method relies on the fact that the size of the grown CNTs is completely governed by the porous length and the number of deposited layers by the CVD conditions [144]. Typically, the oxide barrier layer at the pore’s bottom and the aluminium underneath the PAA film are removed [145]. Therefore, the synthesized CNTs are open from both sides and they are not attached to a layer underneath making them suitable for the synthesis of hybrids structures since the tubes can be filled with molecules [146] or nanoparticles (NPs) [147]. In addition, the carbon nanostructure can easily be modulated by adjusting the diameter of the pores (altering the anodization conditions during the PAA film formation). Consequently, this method is very attractive since the CVD can be performed right after the PAA fabrication avoiding any post-processing step. The two major drawbacks of the catalyst-free method are that the CNTs length is fully constrained by the PAA thickness. To overcome this issue, a plasma-assisted CVD process arises as a solution [148], nevertheless, the CNTs yield is compromised. Furthermore, the crystallinity of the synthesized CNTs is poor [149,150] as can be verified from the Raman spectrum, and only it can be improved by annealing the PAA/CNTs composites at 2800 °C [151]. Due to this, electronic applications are severely limited. Figure 10 summarizes the typical methodology implemented for the non-catalytic synthesis of CNTs inside PAA templates.

**Figure 11:**
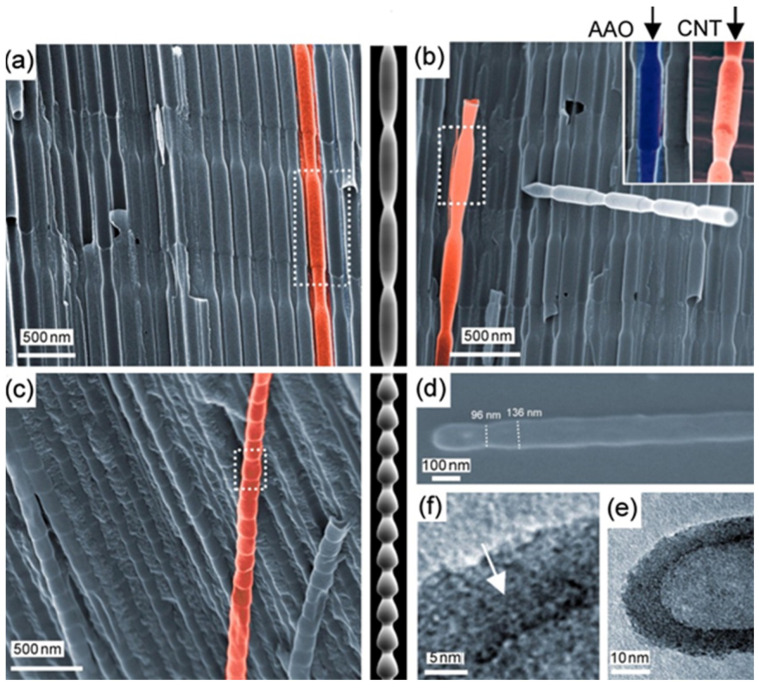
CNTs growth inside PAA templates by a catalyst-free approach with a periodically tailored morphology with (**a**) long, (**b**) middle and (**c**) short segments, this work was performed by the Lusan group. Inset in (**b**) shows the shape of the pore and corresponding CNTs structure. (**d**) Free CNTs with shaped structure and closed bottom after removal of PAA template. (**e**,**f**) TEM images of CNTs. Reprinted with permission from Ref. [153]. Copyright © 2023 Elsevier Ltd. All rights reserved.

There is general agreement that the growth evolution of CNTs within PAA templates follows five different stages [28] characterized by (i) pyrolysis of the carbon feedstock, (ii) carbon dissociation along the deposition zone, (iii) carbon transfer from the source to the PAA template, (iv) carbon nucleation and deposition onto the inner walls of the surface of PAA structure, and (v) the formation of multi-walled CNTs. Then, as the synthesis evolves the CNTs start growing from the pore’s walls towards the pore centre. Dusan Losic’s group carried out very interesting work taking advantage of the PAA versatility to grow CNTs to develop molecule transports applications [28,152]. For instance, Mezni et al. [153] modulate the pore structure altering mild and hard anodization processes to tailor CNTs diameters inside the PAA template, as is shown in Figure 11.

To get a better understanding of the catalytic influence of the PAA film on the growth of CNTs, Alsawat et al. [28] prepared PAA templates with different types of electrolytes. In this way, as described in Section 2.2.2 different impurities levels are obtained close to the pore wall structure. With this, different catalytic activities during the CNTs growth have been detected depending on the anion level. Furthermore, diffusion performance tests of Rose Bengal molecules (a dye molecule model) have been executed to study the impact of the geometry features of the resulting CNTs/PAA composite. Despite the different anion levels on the studied PAA-CNT membranes, the obtained results confirm that the geometric features of the membrane are the most determining factor in the performance of the transport properties of the resulting membranes. Recently, Ryzhkov et al. [154] compared experimental data with gas phase and surface reaction models to describe the growth of CNTs in PAA membranes. In this work, the PAA is fabricated via anodization in a sulphuric acid media, and various parameters involved in the CNT deposition were varied, such as the pressure, temperature, carrier gas flow, and carbon feedstock flow. The performed simulations fairly agree with the experimental results. The authors conclude that the CNT thickness near the membrane top surface is slightly higher than that in the centre region of the membrane. Besides, the carbon growth rate is increased as the synthesis temperature and pressure increase, while it decreases with the carrier gas flow rate. The work performed by Ryzhkov et al. [154] contributes to a better understanding of the CNT grow kinetics inside PAA templates and provides specific guidelines for the synthesis of PAA/CNTs assemblies with a precisely controlled in the nanoscale.

**Figure 12:**
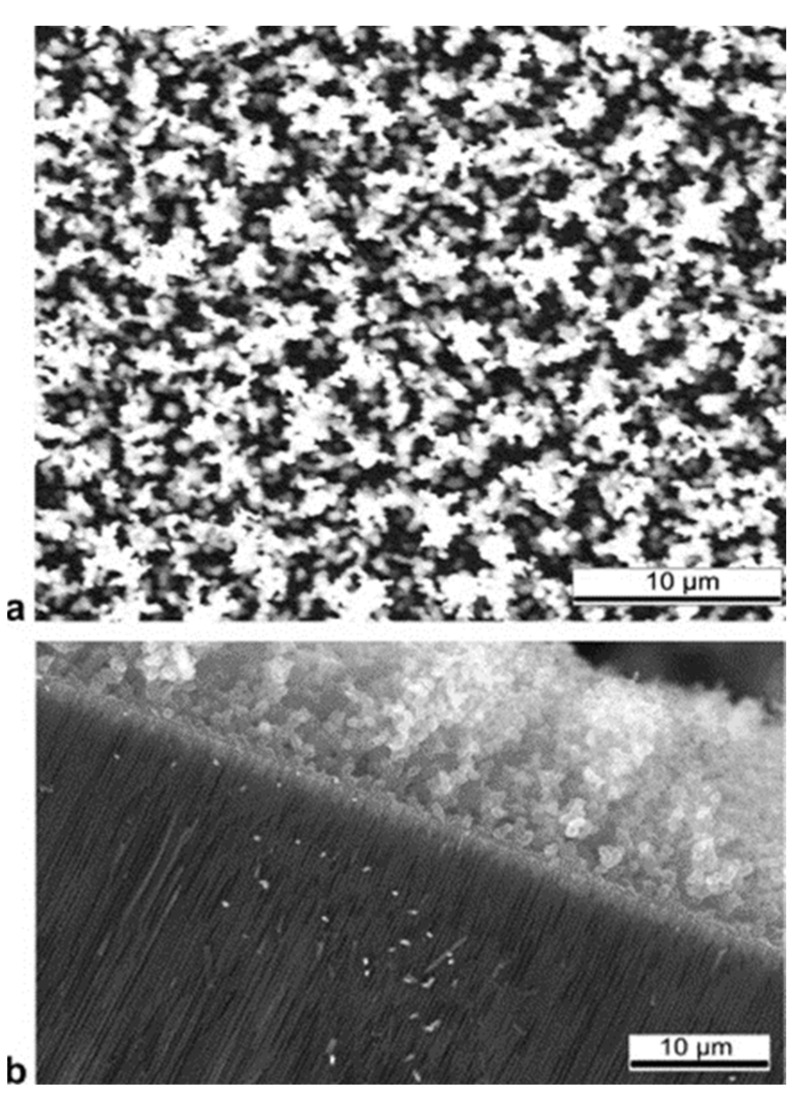
PAA/CNTs composite surface after non-catalytic synthesis of CNTs. (**a**) Top view and (**b**) side view where is identified a 20 μm thick amorphous carbon overgrowth layer on top of the PAA surface. Reprinted with permission from Ref. [144]. Copyright © 2023 Elsevier Ltd. All rights reserved.

It is important to point out that the hydrocarbon nucleation also takes place at the top surface of the PAA film. Many researchers reported [144,155,156] that the CNT growth is accompanied by deposition of amorphous carbon on such surfaces, as Figure 12 indicates. This carbon film is difficult to remove by chemical approaches and may induce a major drawback towards the development of PAA/CNTs based applications. Scheiner et al. [144] proposed a gas-phase/solution method to obtain large-area (of the orders of cm^2^) free of amorphous carbon on the surface of a PAA/CNTs assembly. However, the applied methodology entails some mechanical damages on the CNTs network that does not allow further filling or infiltration inside PAA/CNT composite. Interestingly, Mezni et al. [151] develop a methodology to suppress the a-C where the carbon feedstock is introduced in cycles, combining steps with the hydrocarbon followed by a rest cycle where just a carrier gas is flown.

In summary, the template approach to fabricate PAA/CNTs composites is very elegant due to their relative ease of implementation, the precise control of the CNT morphology, and the preservation of the collectively CNTs organizations (the tubes cannot emerge from the pores). Despite the poor crystalline structure, this platform is very attractive for further functionalization since both tubes’ ends are opened.

#### 3.1.2. Synthesis of CNTs within PAA Templates Using Catalyst NPs

CNTs are generally grown using a metal [157]/metal oxides [158,159] catalyst on a substrate. The catalyst/substrate interaction plays a major role in the synthesis of CNTs obtained by the CVD method. Different characteristics can be determined through the catalyst, such as the diameter, yield, CNTs quality, and even more. Regarding SWCNTs, many studies are focusing on nanotube growth with a specific chirality by tuning the catalyst [160,161]. Recently, Esteves et al. [162] reported an interesting review detailing the state-of-the-art of carbon nanotubes and/or CNFs synthesized via a CVD process, particularly pointing out the parameters governing the carbon nanostructure synthesis.

**Figure 13:**
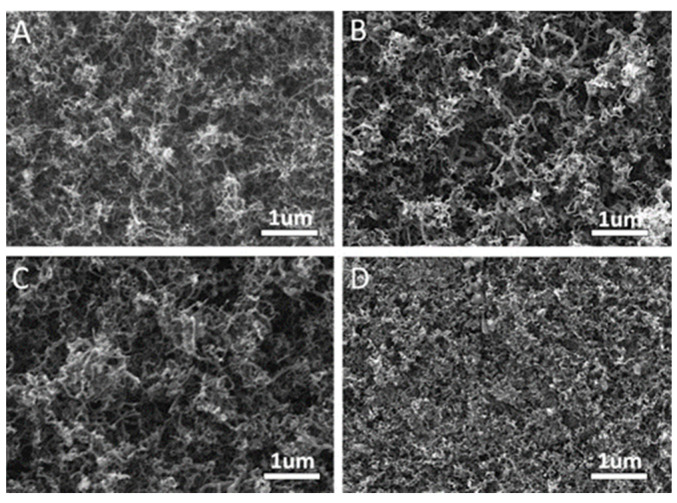
SEM images of the PAA/CNTs after CVD process using different Ni catalyst contents: (**A**) 0.1 molL^−1^ Ni content, (**B**) 0.5 molL^−1^ Ni content, (**C**) 1 molL^−1^ Ni content, and (**D**) 2 molL^−1^ Ni content. Reprinted with permission from Ref. [163] Copyright © 2023, American Chemical Society.

The implementation of PAA as substrates implies that catalyst deposition requires a multi-step process. Over the years, many techniques have been proposed to deposit catalytic NP within the pores. The first work reporting the synthesis of CNTs inside PAA templates using a catalyst was carried out by Che et al. [26], in this case, 0.1 M Fe(NO_3_)_2_ or Co(NO_3_)_2_ were immersed inside the PAA templates, and the solutions were annealed under an inert atmosphere at 400 °C. This deposition method may lead to a NP distribution along with the pores, with catalyst diameters smaller than the pore’s size. The catalyst is not confined within the porous structure, under this configuration, after the CVD process, an entangled CNT distribution is obtained. Similar results regarding the CNTs distribution emerging from the porous structure were obtained from Liu et al. [163] who also adopted an immersion method, using nickel nitrate hexahydrate dissolved in ethanol, a representative image of CNTs emerging the PAA is shown in Figure 13. It is worth noting that Liu et al. [163] used waste plastics as feedstock for the CVD synthesis to grow the CNTs. To verify the distribution of the formulated NPs inside the porous structure, a cross-section analysis is required. A powerful approach consists of a lamella preparation and TEM observation that allows obtaining an accurate physical/chemical description of the NPs distribution inside the pores in a cross-section perspective. The lamella preparation can be prepared by means of Focused Ion Beam (FIB) and further analyzed by advanced TEM characterization techniques such as STEM-EDX and EELS spectroscopy [83,164].

**Figure 14:**
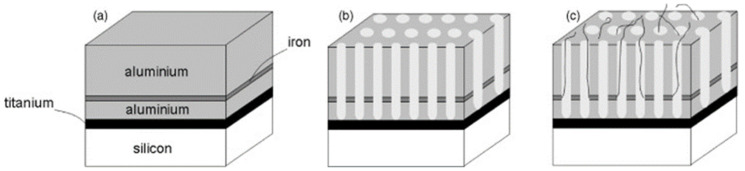
Schematic description of catalyst preparation inside PAA and CNT synthesis procedure. (**a**) Initial deposited film structure. (**b**) Anodized film structure. (**c**) CNTs synthesized from pore channels. Reprinted with permission from Ref. [11]. IOP Science Copyright © 2023.

**Figure 15:**
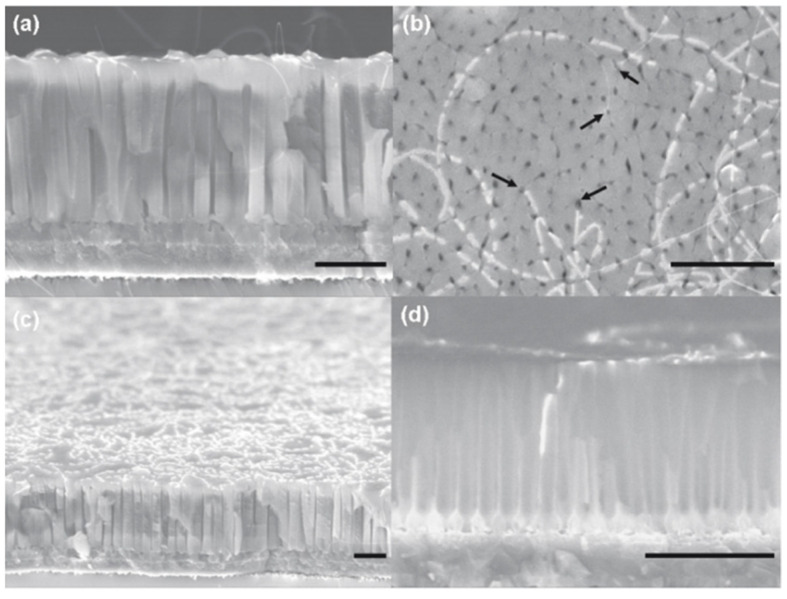
SEM images of CNTs grown inside the PAA template. (**a**) Cross-sectional view of PAA/CNTs. (**b**) Top PAA surface showing CNTs emerging from pores. (**c**) Tilted cross-sectional view of template and CNTs. (**d**) Cross-sectional view showing CNT initiating from catalyst layer. Scale bar = 500 nm. Reprinted with permission from Ref. [11]. IOP Science Copyright © 2023.

Interestingly, Maschmann et al. [11] proposed a method based on electron beam evaporation of different thin films (titanium, aluminum and iron), as described in Figure 14. After a SiO_2_ deposition for adhesion purposes, a stacked structure of Ti/Al/Fe/Al, as indicated in Figure 14a, was deposited onto a Si wafer. A first anodization process was carried out on the top layer and further removed to execute the two-step anodization process. The second anodization process was carried out up to the Ti layer, as shown in Figure 14b. The authors pointed out that after the anodization process, the Fe catalyst metal was locally embedded into the pore wall structure. This contrasts with other thin films such as Ni, Co, and Pd where a delamination phenomenon occurs during the anodization process. The CNTs synthesis, as sketched in Figure 14c, was performed by microwave plasma-enhanced chemical vapor deposition (PECVD) [165]. Figure 15 shows a representative CNTs distribution obtained by the proposed approach. Many CNTs arise from the top PAA surface forming CNT bundles. The HRTEM and Raman spectroscopy analysis revealed that a mixture of SWNT and double-walled carbon nanotubes (DWNTs) are synthesized inside of the PAA templates. This is a great achievement for the PAA/CNTs assemblies.

It is well known that the synthesis of SWCNTs requires catalyst sizes of the order of a few nanometers [166], regarding the PAA templates, is very challenging to control catalyst deposition after the template fabrication in the nanometer scale because the pore size is typically larger than 10 nm [31]. Furthermore, the temperatures involved in the synthesis of SWCNTs are generally carried out at temperatures higher or close [167] to the aluminium melting point (655 °C). Therefore, to perform the CVD synthesis at temperatures above 600 °C is necessary to fully remove or completely anodized the aluminium underneath the PAA film. The approach introduced by Maschmann et al. [11] overcame these two issues by depositing a thin film layer between the aluminium and subsequently anodizing the full aluminium layer. To the best of our knowledge, this is the only approach where the catalyst deposition is performed before the PAA preparation. Fisher’s group intensified the research on this approach by performing a parametric study focused on the CNTs synthesis from catalytically active PAA anodized from Al–Fe–Al multi-stacked thin layers [168], to analyse the influence of the pore aspect ratio, Fe layer thickness, CNT synthesis temperature, and pre-anodization thermal annealing towards the optimization of template CNT synthesis.

The electrodeposition technique is an attractive route to deposit metal NPs at the bottoms of the pores. The electrodeposition process is characterized by its low cost of implementation, straightforward utilization, ambient operating conditions, and the capability to control the properties of deposited materials such as size and density by adjusting the parameters involved in the deposition process. Compared with other chemical routes, the electrodeposition method scalability is not constrained by the slow kinetics and the other batch operation techniques which results in undesirable variability of NP physicochemical properties [169]. Furthermore, a wide variety of metal [170,171], metal-oxides [172,173] and semiconducting [174] structures have been successfully electrodeposited at the bottom of the pores. It is important to point out that the most common metal catalysts for the synthesis of CNTs, such as nickel [175], iron [176], and cobalt [177] have been electrodeposited within PAA. The implementation of the electrodeposition process inside porous templates is not limited to catalyst deposition but is also vastly used for the synthesis of other nanowires [171] or nanotubes [178], proposed for a wide range of applications [179]. Besides, the same experimental setup used for the anodization process can be used for the electrodeposition method. Then, the electrodeposition technique is one of the best bottom-up approaches to homogeneous deposit nano-porous templates. The critical structural parameter from the PAA template that determines the electrodeposition process is the oxide barrier layer at the pore’s bottom. The barrier dielectric layer imposes a potential that must be overcome to reduce the electrolyte solution in the electrochemical bath. For further details of the parameters involved in the electrodeposition process, the reader can refer to the review paper from Sousa et al. [180], which also includes a detailed explanation of the different electrodeposition modes (DC electrodeposition, AC electrodeposition and Pulsed electrodeposition). In consequence, the templates must be appropriately tailored to guarantee good electrical contact between the electrolyte that fills the pores and the layer at the bottom of the pores, only then the reduction of electrolyte cations can occur using a potentiostatic or galvanostatic process. 

Different methods have been developed to reduce or remove the oxide barrier layer at the bottom of the pores. For instance, aluminium can be evaporated/sputtered into a conductive substrate and subsequently the anodization process is performed until the underlying conductive support is reached [150,181]. However, it is necessary to consider that for PAA templates fabricated by a two-step anodization technique, a thick sacrificial layer is necessary to pre-texture the aluminium surface. However, the evaporated/sputtered aluminium thickness typically is in the order of micrometres [182]. Subsequently, the regularity of the pores can be severely reduced since the pore structure cannot reach a honeycomb-like structure at the end of the first anodization process. Gras et al. [183] grew CNTs inside the alumina matrix templates adopting the explained methodology and further Ni electrodeposition. The PAA pore ordering degree is relatively low. But their work reveals strong confinement of the nanopore walls and the synthesized CNTs.

Similarly, to eliminate the oxide barrier layer, there is a method that consists of a membrane fabrication (obtained by anodization step and further fully etch the pore’s bottom) and subsequently metal evaporation/sputtering [184,185]. This procedure is not affected by the aluminium thickness that influences the pore ordering degree when a two-step anodization process is performed. However, regarding the CNTs synthesis, the metal evaporated/sputtered can be embedded inside the pores that can potentially affect a further CVD process. To avoid this problem, metal with non-catalytic activity has to be deposited, as Angelucci et al. demonstrated by using a 100-nm-thick Cr layer followed by a 150-nm-thick Au layer as a conductive layer. In this method, PAA membrane manipulation is required, which is not desirable for a potential scale-up. Reactive ion etching (RIE) [186,187] has also successfully been implemented to remove the cap from the bottom pores without triggering pore widening effects associated with wet chemical routes. There is no report of PAA/CNTs/Catalyst using RIE to dissolve the oxide barrier layer and further electrodeposited catalytic NPs for the synthesis of CNTs. 

Alternatively, there is a procedure that can be executed in the same electrochemical bath as the anodization process and started at the end of the anodization process. This process is called the barrier layer thinning method (BLT) developed by Furneaux et al. [96]. The BLT consists of a gradual decrease in potential after the desired PAA thickness is reached. The idea is to exploit the linear dependence between the applied potential and the barrier dielectric layer at the bottom of the pore, see Equation (3). The potential rate decrease can highly influence the PAA structure. After each potential drop, the electrochemical system is self-adjusted to a new equilibrium with an associated new cell structure following the dependence of the structural parameters expressed in Equations (1)–(3); however, to reduce the oxide barrier layer, the electrochemical system is gradually perturbed. Then, the whole process in each potential decrease enters a new equilibrium condition leading to a non-steady-state anodization process. In case the potential drop is too fast, the ongoing anodization process can be interrupted.

Cheng et al. [188] provide a detailed explanation about the formation of the PAA under a non-steady-state anodization process. It is important to highlight that the BLT can lead to the formation of a tree-like branched structure known as dendrites [189]. Some studies were focused on the characterization and control of these structures. Besides, the barrier thinning at the pores’ bottom this methodology can be implemented to fabricate reproducible hetero-structures with a convenient adjustment of the anodization conditions [190,191,192,193]. Li et al. [194] set the guidelines to growing individual Y-junction nanotubes based on PAA template growth, where the Y-junction was obtained by reducing the potential by a factor of $1/\sqrt{2}$ in the ongoing anodization process.

**Figure 16:**
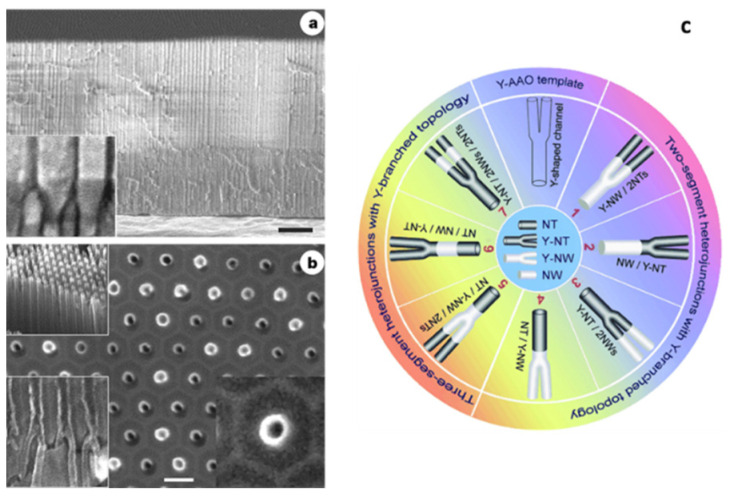
(**a**) SEM cross-section of a Y-branched template obtained by reducing the anodization by a factor $1/\sqrt{2}$. (**b**) SEM images of the branched PAA template after CNTs synthesis using electrodeposited Co catalyst. (**c**) Catalogue of CNT/NW and NT/NW/NT hybrid nano-architectures. CNTs (hollow) are represented in black and NWs (solid) in white; Y-NT and Y-NW indicate Y-shaped NTs and NWs. Reprinted with permission from Ref. [194]. Copyright © 2023 WILEY-VCH Verlag GmbH & Co. KGaA, Weinheim, Germany.

Figure 16 summarizes the kind of hetero structure that can be engineering applying a BTL process. In particular, Figure 16a shows the cross-section of a Y-branched template consisting of a 3 mm long primary pore and their respective two secondary ‘branches’ of 2 mm. Figure 16b shows SEM images of the branched PAA template after CNTs synthesis using electrodeposited Co catalyst, with a remarkable high CNT yield inside the PAA template. Figure 16c summarizes a schematic catalogue proposed by Meng et al. [193] of the compatible heterojunctions based on nanowires/nanotubes obtained by exploiting the versatility of the PAA fabrication altering the anodization potential combined with electrodeposition process and/or further CNTs synthesis.

**Figure 17:**
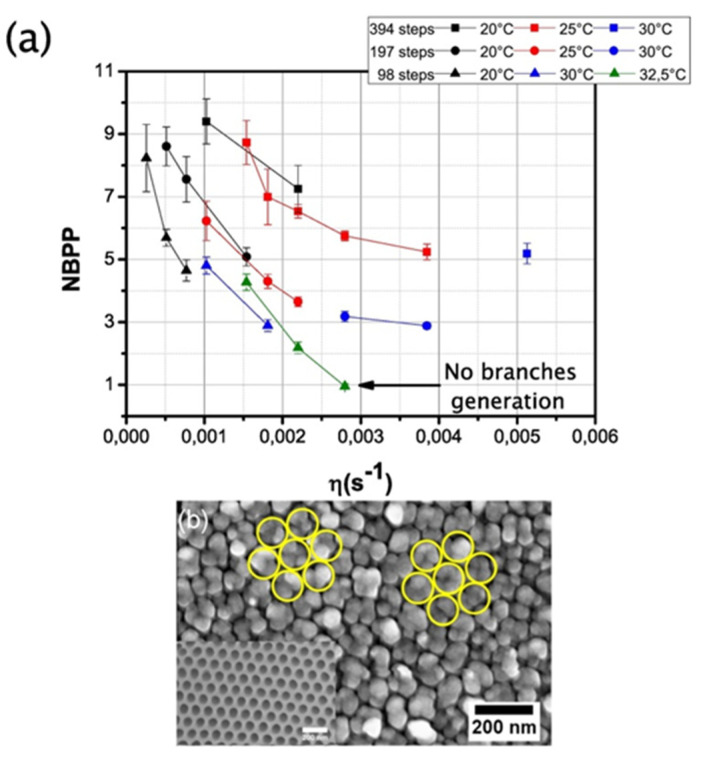
(**a**) Number of Branches generated by Primary Pore (NBPP) as a function of the exponential potential decay rate at different anodization temperatures and various number of voltage steps. (**b**) SEM micrograph showing the Ni NPs deposition after the PAA removal via a chemical etching. The PAA template has been prepared using the condition for which no branch formation is expected. The anodization temperature was set at 32.5 °C, the total number of steps was 98, and the exponential voltage decay was 2.28 × 10^−3^ s^−1^. In yellow is pointed out the former pores’ positions. Reprinted with permission from Ref. [100]. Copyright © 2023 Elsevier Ltd. All rights reserved.

Regarding the reduction of the oxide barrier, precise control of the potential decrease is required for a reproducible PAA template. For this purpose, the potential decrease rate highly influences the structure at the pores’ bottom. Typically, the potential stepwise is decreased at 5% of the previous anodization potential value, up to a minimum value of 0.3 V [96,195]. In a previous work [100], the influence of the voltage decrease rate and the anodization temperature was implemented to analyse the porous tree-like branched structure. The adopted approach to quantify the number of branches created per primary pore (NBPP) was based on a combination of a pulsed-electrodeposition (PED) process applied to fill the nano-pores bottom, and subsequently, remove the PAA film. The obtained electrodeposited NPs distribution reflects the fingerprint of the previous shape/state of the bottom of the pores. Extrapolating the NBPP can be obtained simply comparing the density of pores over the density of NPs [100]. This methodology was implemented since a direct observation of the branched structure by SEM is not accessible. On the other hand, TEM analysis implies time-consuming lamella fabrication and provides very localized information of the sample. Figure 17 shows a summary of the NBPP obtained for different anodization conditions, because of this study was demonstrated that PAA templates with straight pores up to heterostructures PAA templates with 10 branches.

**Figure 18:**
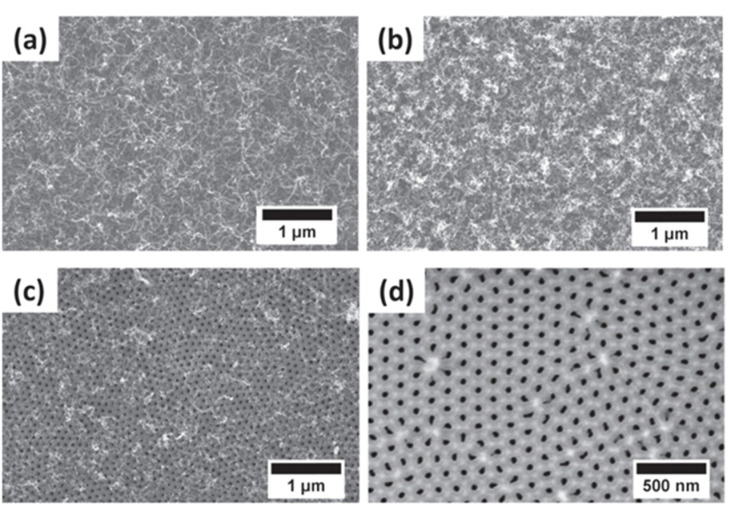
SEM micrographs of PAA templates fabricated with different Number of Branches generated by Primary Pore (NBPP) and subsequently Ni electrodeposition and CNTs synthesis. (**a**) 2, (**b**) 3, (**c**) 6 and (**d**) 7 NBPP. Same CNT synthesis conditions were applied for all of the PAA/Ni composites. Reprinted with permission from Ref. [83]. IOP Science Copyright © 2023.

**Figure 19:**
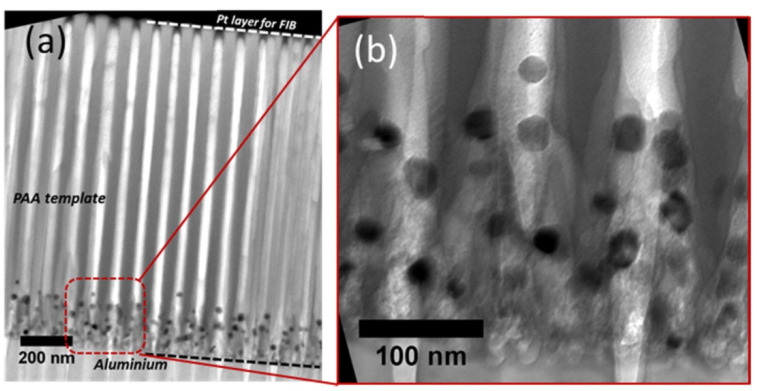
TEM observation of the FIB cross-section lamella of a PAA template with an average of 6 NBPP where synthesis of the CNT was carried out: (**a**) a low magnification TEM image showing the whole porous structure with the NP localized at the branched zone. (**b**) Magnification on an area of the bottom pores evidencing CNTs couldn’t evolve through the PAA template. Reprinted with permission from Ref. [83]. IOP Science Copyright © 2023.

The presented branched structure can have a dramatic consequence on the CNT synthesis [83]. Figure 18 summarizes different PAA templates where an exponential voltage decrease process was applied to partially dissolve the oxide barrier layer at the bottom of the pores and Ni catalyst was subsequently deposited by a PED process. Finally, a CNTs synthesis was performed. It is observed that for both samples with two and three NBPP, Figure 18a,b respectively, a typical spaghetti-like distribution covers the entire PAA template surface. As the NBPP increase up to six, the CNT amount drastically decreases, as reflects Figure 18c. This is supported by a TEM image with an insight at the bottom pores zone as shown in Figure 19. For a PAA template with an average of eight NBPP, no CNTs were found to emerge from the pores. By a lamella preparation and further TEM observations, it was established that not only the number of branches but also the catalyst size determines the growth evolution inside the pores. The CNTs that arise from the pores are those in which the NP diameter and pore diameter are similar. In this case, no competition between CNT is established inside the pore, and the tubes grow as the carbon molecules reach the catalyst. In contrast, when more than one tube can be catalyzed from the metallic NP a competition is established inside the pore and the pore can become clogged by the CNTs. Ke et al. [196] also performed a study of the influence of the catalyst size on the CNTs growth inside the PAA templates, but this work was mainly focused on Co NPs with a significantly smaller size than the pore host. The authors succeed to control the Co NP size by adjusting the electrochemical parameters; however, further optimizations must be performed uniform deposition of Co NPs of any desired size that will lead to homogenous CNT growth.

In summary, the BLT technique can be described as a well-designed approach since the process is executed in the same electrochemical cell than the anodization process and the pore structure can be easily tailored adjusting the anodization parameters. However, the geometrical features can affect the growth evolution inside the pores.

A variation of the potential decrease method is the so-called cathodic polarization introduced by Zhao et al. [197]. After the steady-state anodization process, an exponential potential decrease process is applied followed by a cathodic polarization performed in a 0.5 M KCl neutral solution with an underlying aluminium layer of the PAA as the cathode and a graphite plate as the anode. The main constraint of the cathodic approach is that the effectiveness to dissolve the barrier layer depends on PAA film thickness and is proven to be more effective in relative thin PAA templates. This methodology has not yet been explored for CNTs growth inside PAA templates. 

In conclusion, many approaches have been proposed to the growth of CNTs inside PAA templates using metal NPs catalysts. Generally, this method requires extra steps with respect to the catalyst-free method making the PAA/CNTs composite fabrication more complex. However, the grown carbon nanostructures are less defective, and the tubes can emerge from the pores that can be interesting for some applications, as will be described in Section 4.

### 3.2. Graphene and PAA

The successful isolation of monolayer graphene sheets by mechanical exfoliation from bulk graphite launched the field of two-dimensional (2D) materials to another level [198]. PAA templates can offer a suitable platform to develop different graphene applications. However, few works reported the graphene synthesis within PAA templates; rather, a 3D graphene was synthesized using the PAA as a host matrix [199,200]. Herein, graphene is referred to as a single-layer or multi-layer of atoms arranged in a two-dimensional honeycomb lattice, not rolled-up to form a hollow cylinder equivalent to a CNT. 

**Figure 20:**
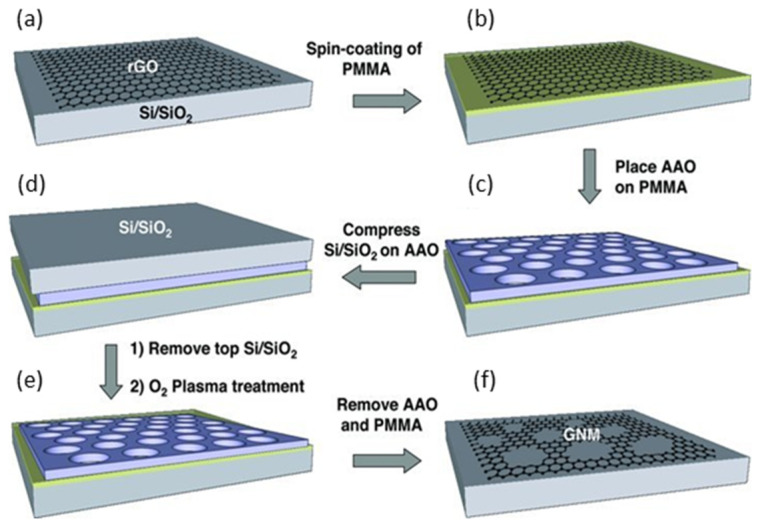
Schematic illustration of the route developed by Zeng et al. [201]: graphene nanoribbons are obtained using an etching mask of PAA. (**a**) Graphene transfer on a Si/SiO_2_ substrate, (**b**) Spin-coating of PMMA, (**c**) Placing of PAA template on PMMA, (**d**) Compression using Si/SiO_2_ substrate, (**e**) Plasma treatment to etch the graphene, (**f**) removal of the PAA and PMMA film. Reprinted with permission from Ref. [201]. Copyright © 2023 WILEY-VCH Verlag GmbH & Co. KGaA, Weinheim, Germany.

In most of the reported studies, PAA was used as a mask where a transfer process is involved. A representative work on this topic was performed by Zeng et al. [201] where they fabricate graphene nanoribbons using a PAA layer as a hard etching mask under an O_2_ plasma treatment, as is shown in Figure 20**.** The proposed approach avoids the utilization of reactive ion etching based on toxic gases, such as CHF_3_ and CF_4_. Regarding nanoelectronics applications, this method is attractive to develop graphene nanoribbon-FET based devices since CHF_3_ and CF_4_ can also etch the Si/SiO_2_ substrate affecting the gate modulation during the FET operation. Inspired by this procedure, Lee et al. [202] fabricated FETs using large-scale nanoporous graphene. The authors used different PAA masks with different pore sizes by simply varying the pore widening time but no details are given explaining the influence of changing the neck width on the electrical characteristics of graphene.

**Figure 21:**
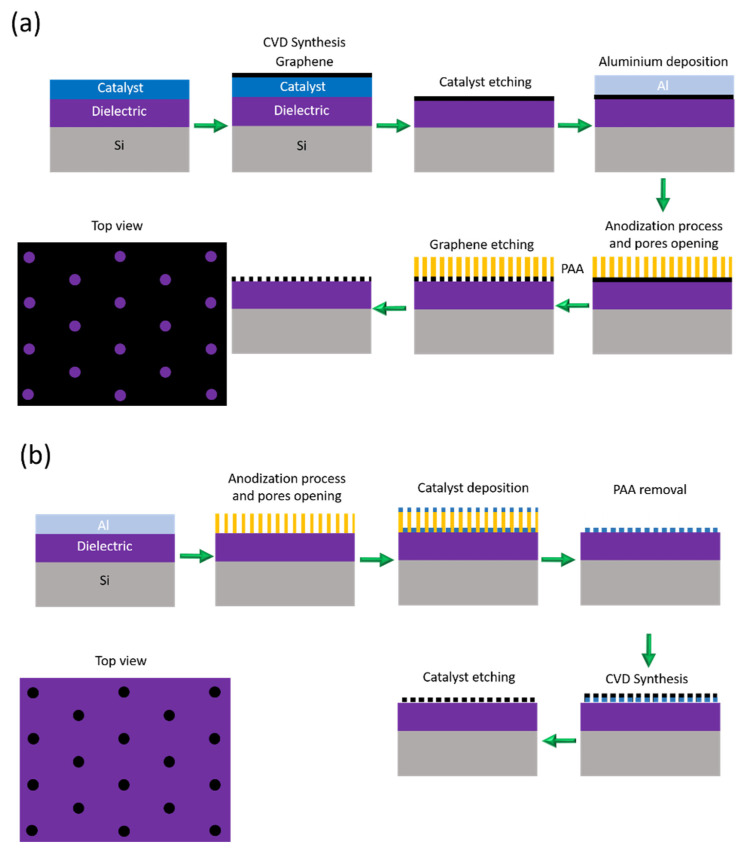
Schematic representation of workflow proposed to fabricate a PAA/graphene assembly with a transfer-free process. (**a**) The workflow to obtain a positive pattern of graphene on a substrate. (**b**) The workflow to obtain a negative pattern of graphene on a substrate.

It could be interesting to explore the possibility of fabricating graphene/PAA structures using a transfer-free approach [203,204]. In the present review, we proposed some strategies to exploit the benefits of the PAA as a mask and avoid the limitations of the exfoliation and further transferring process. Figure 21 summarizes possible routes to fabricate graphene-based devices using a transfer-free process. Positive and negative graphene patterns (with respect a PAA mask) can be obtained on a dielectric layer. For instance, Figure 21a illustrates the procedure to obtain a positive pattern with a transfer-free approach. First, the graphene is grown by a CVD process, subsequently, the graphene is released from the catalyst [205], then, aluminium can be sputtered or evaporated on the graphene and afterwards the anodization process can be performed until the graphene is reached, followed by wet etching to open the pores. At this stage, the PAA act as a mask similarly as in Zeng et al. [201], therefore, an O_2_ plasma can be used to etch the exposed graphene and finally, remove the PAA via wet chemical etching. Alternatively, to obtain a negative pattern, as schematized in Figure 21b, the aluminum metallization must be performed onto the graphene catalyst, then, the PAA fabrication must be performed, removing the oxide barrier layer at the bottom of the pores. Subsequently, the graphene synthesis can be executed, followed by the PAA removal and the graphene release. Both kinds of graphene patterns can be attractive for further functionalization taking advantage of a large number of active sites present on the edges.

Regarding the direct synthesis of graphene within PAA, Zhan et al. [199] developed three-dimensional (3D) graphene conformably coated on porous structure via PECVD. The authors compared the Raman spectrum of a graphene-coated PAA and a diamond-like amorphous carbon-coated PAA composite. The authors found that the plasma-assisted route leads to a better graphene quality in terms of the density of defects on the graphene crystal lattice than a simple thermal graphitization process. This conclusion was obtained by identifying the typical graphene related intensity peaks of graphene D (1348 cm^−1^), G (1595 cm^−1^) and 2D (2691 cm^−1^) peaks, and calculating the intensity ratio between D and G peaks, that has been used as a standard method to measure of inter-defect distance in graphene. This kind of compound can be very interesting to develop for electrochemical applications. 

There are some applications published using a PAA/graphene assembly [206,207,208,209] but in all of these cases a graphene transfer process is involved. This step can complicate the fabrication and induce reliability and reproducibility issues. On the other hand, there is not any reported work focused on the direct planar graphene synthesis on the PAA template. Consequently, there is an interesting challenge and opportunity for the materials science community to develop applications based on transfer-free graphene/PAA assemblies.

### 3.3. Diamond-like Carbon (DLC) and Nanodiamond Synthesis within PAA Templates

Diamond and diamond-like carbon (DLC) are of great interest due to their high chemical stability and excellent physical properties [210] for coatings material. Furthermore, due to their marvellous biocompatibility property, these materials are suitable to develop bio-implants applications [211]. On the other hand, PAA-based biomedical applications are not fully exploited because still there is a lack of evidence about the use of alumina in biotechnology. A recent review by Davoodi et al. [212] is devoted to shedding light on the bio-medical potential applications based on PAA. This review article concludes that further studies are needed to prove that PAA is a biocompatible material. Considering that top-down fabrication techniques for diamond-like applications are severely limited due to the high hardness and chemical stability characteristic of the sp3 carbon, renders PAA/DLC composites very attractive since the PAA can be easily tailored. This makes them suitable for a bottom-up approach to fabricate porous nanodiamond and other DLC based materials. 

**Figure 22:**
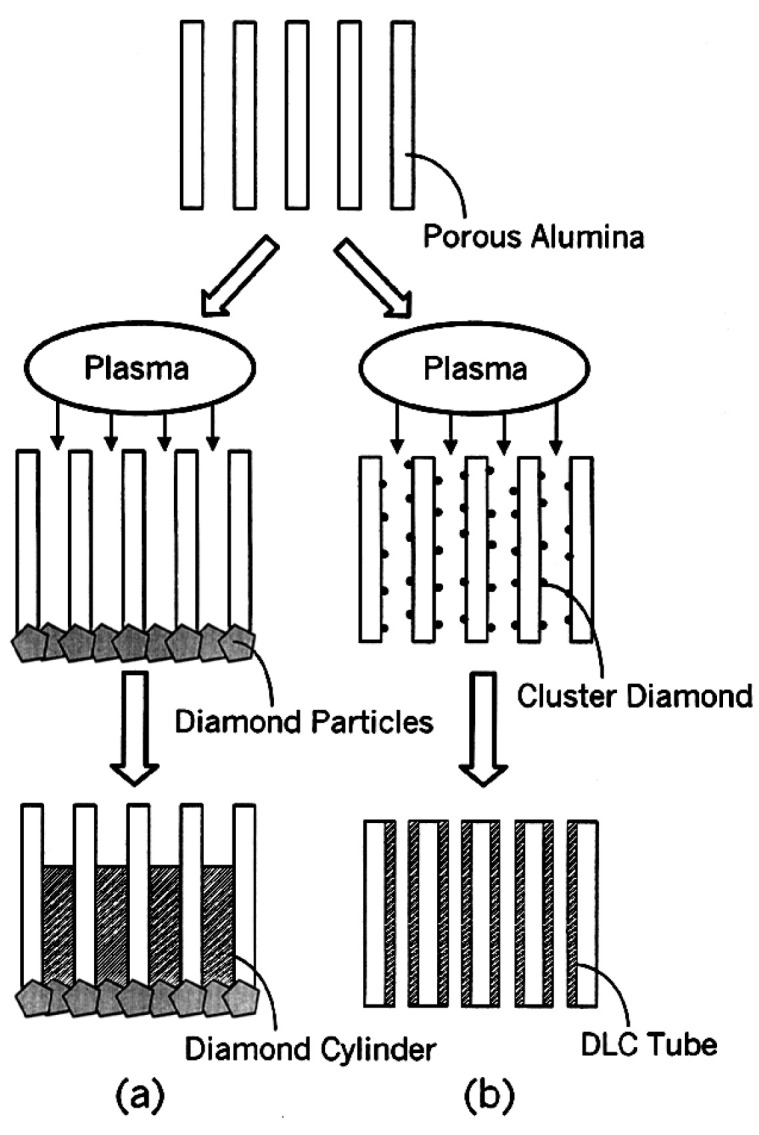
Schematic diagram showing the route for the fabrication of diamond nanostructures within PAA templates proposed by Masuda et al. [213]. (**a**) Diamond NPs with the same pore size deposited at bottom of the pores to synthesize diamond nanocylinders, and (**b**) smaller NPs than the pore size dispersed in an ultrasonic bath to nucleate diamond-like carbon (DLC) nanotubes. Reprinted with permission from Ref. [213]. Copyright © 2023 WILEY-VCH Verlag GmbH & Co. KGaA, Weinheim, Germany.

Synthesis of well-aligned diamond nanocylinders within PAA templates was pioneered by Masuda et al. [213] by microwave plasma-assisted CVD. Two different diamond-type of nanostructures were nucleated depending on the diamond NPs deposition. Diamond NPs were deposited with the same pore size to nucleate diamond nanocylinders. For the growth of DLC nanotubes, smaller NPs than the pore size were deposited immersing the PAA in an ultrasonic bath containing a diamond-based solution. Figure 22 summarizes both approaches to nucleate diamond nanocylinders (Figure 22a), and DLC nanotubes (Figure 22b). A similar strategy was implemented by Yanagishita et al. [214] to nucleate diamond cylinders with square and triangular cross-sections. It is important to highlight that the highly ordered PAA templates with square and triangular pore cross-sections were engineered using a SiC mould with a patterned array of convex to imprint an aluminium foil. Subsequently, the nanoimprints act as preferential sites for the pore nucleation during the anodization step.

New insights regarding the growth mechanism of hybrid diamond and amorphous DLC coated on PAA has been provided by Aramesh et al. [215]. In this study, three different procedures are carried out to fabricate hybrid diamond and amorphous carbon-coated alumina membranes using as a substrate PAA templates. The first approach consists of the exposure of the PAA under hydrogen/methane or argon/methane PECVD to coat the PAA surface with a DLC layer. The other two methods are based on treatments of hydrogen/methane or argon/methane PECVD on PAA samples previously immersed in a NPs dispersion of nanodiamond in water. Based on a set of experimental observations such as Raman spectroscopy, SEM, scanning transmission electron microscopy (STEM), electron energy-loss spectroscopy (EELS), X-ray photoemission spectroscopy (XPS) and near-edge X-ray absorption fine structure (NEXAFS) spectroscopy, the authors demonstrate that the interplay between internal and external carbon supply is a critical factor for the formation of the ultrathin sp3-bonded carbon layer in the nanopores.

**Figure 23:**
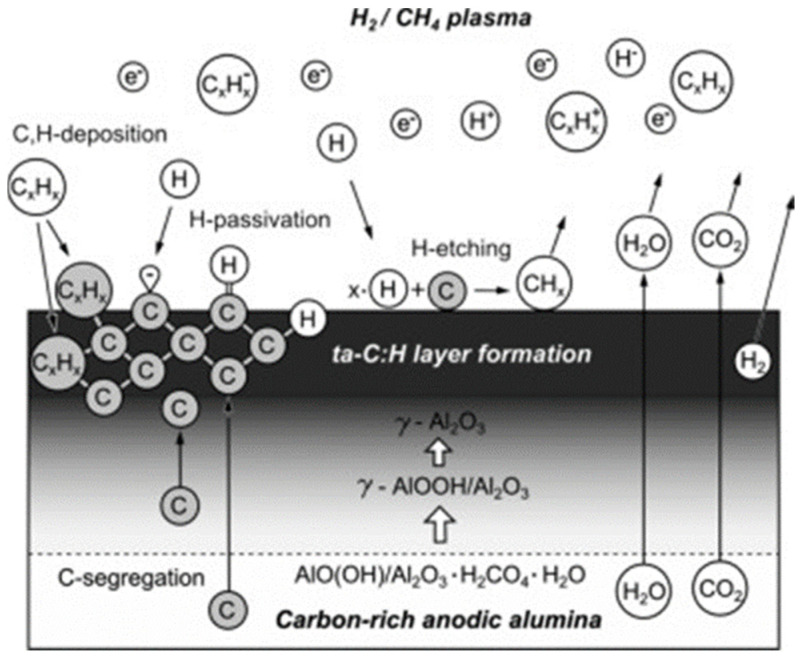
Schematic representation summarizing the growth and nucleation of the ultrathin DLC layer inside and within the alumina surface as proposed by Aramesh et al [215]. Reprinted with permission from Ref. [215]. Copyright © 2023 Elsevier Ltd. All rights reserved.

Figure 23 shows a schematic representation of the growth and nucleation mechanisms of the ultrathin DLC carbon at the alumina surface. The authors proposed three distinct steps to self-limited the DLC growth mechanism. Basically, during the first step, the plasma treatment leads to a segregation of water, CO_2_, and subsurface carbon hydroxyl functional groups. This carbon content is related to residues from the PAA formation due to the anodization process [216]. Then, the carbon content acts as nucleation sites for the growth of DLC, and the hydrocarbon, energetic carbon and hydrogen species supplied by the plasma leads to the formation of an amorphous carbon layer. The carbon incorporation is stopped when there are no more active sites remaining and finally, the DLC growth is spontaneously finished when the carbon supply from the subsurface is complete.

Different plasma treatment durations have been applied (ranging from 10–60 min) and the DLC film thickness remains constant. Interestingly, the authors verify the superior permeability and material quality resistance of the DLC/PAA assembly in various strong acidic and alkali environments in which pristine anodic alumina are dissolved, demonstrating the effectiveness of the DLC coating layer against corrosive chemicals. Therefore, many technological applications can be exploited using hybrid diamond and DLC coated on PAA [217,218,219].

Overall, the PAA templates are an interesting platform for synthesized diamond-based materials. The control of the pore cell structure enables to tailor diamond structures by a bottom-up approach avoiding complex and difficult etching techniques [220]. Hybrid diamond and DLC coated nanoporous alumina materials exhibit promising properties for implants applications. However, more effort must be performed to fully prove the biocompatibility of PAA.

### 3.4. Other Carbon Nanoforms within PAA Templates

A wide diversity of carbon allotropes, like CNFs, amorphous carbon structures, platelet nanostructures, carbon nano-belts and carbon dots have been synthesized inside the PAA templates, due to the previous advantages that the ordered PAA can provide with confined and tuneable nano-channels. These PAA/Carbon-based materials are interesting platforms to develop energy storage applications, sensing devices and composite materials. 

**Figure 24:**
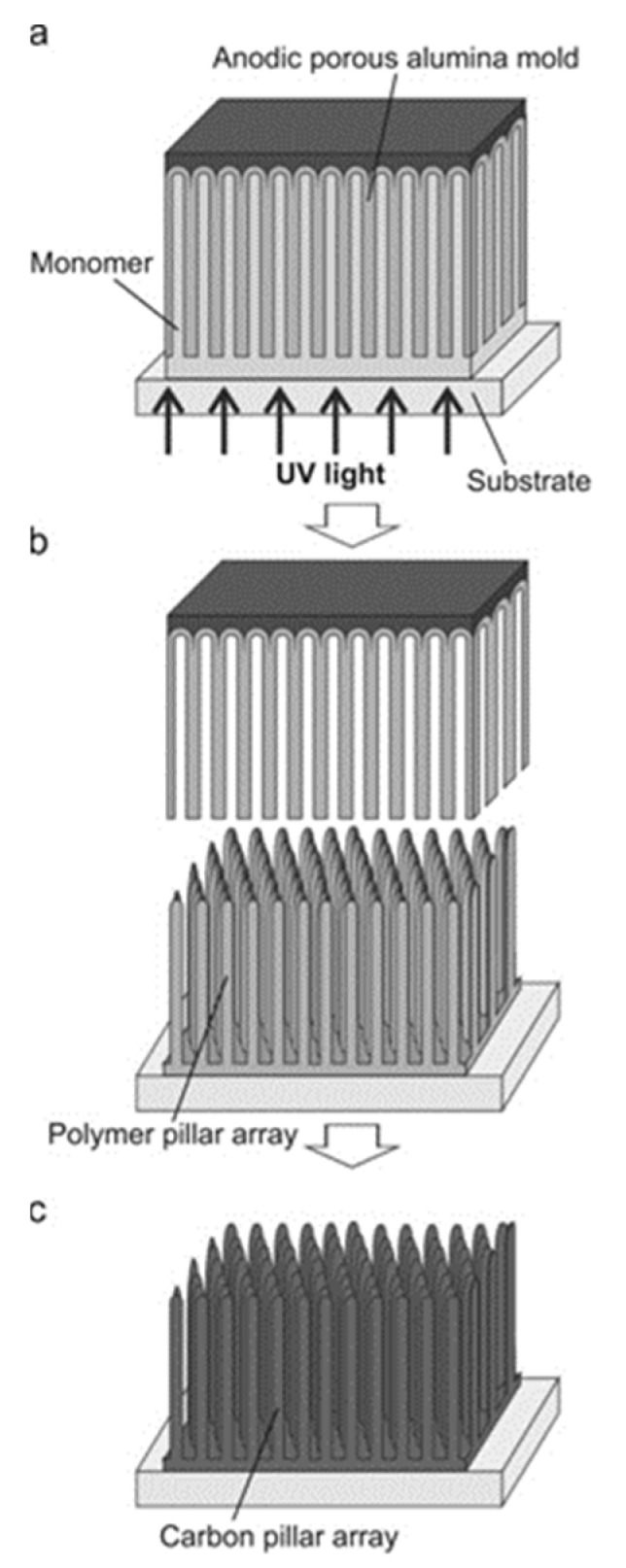
(**a**) Schematic representation of steps involved in the fabrication of an array of CNF with a high aspect ratio by nanoimprinting using PAA as a mold: (**a**) nanoimprinting, (**b**) detaching the mold, and (**c**) carbonization of a polymer by heat treatment under vacuum. Reprinted with permission from Ref. [222]. Copyright © 2023 Elsevier Ltd. All rights reserved.

Taking advantage of the tubular structure of PAA templates, many researchers focused on the synthesis of CNFs inside PAA templates. Similarly, as for CNTs, both catalyst-free and the deposition of catalytic NPs approaches were implemented. For the template method, different precursors were explored such as polycyclic aromatic hydrocarbons [221], polymers [222,223], or acetylene pyrolysis [224]. Alternatively, CNF arrays from high-aspect-ratio polymer pillars were fabricated by T. Yanagishita and H. Masuda [222]. In this case, the PAA template was used as a mould, where a monomer was added and further polymerized by UV irradiation. The obtained polymer nanofiber array was achieved after detaching the mould from the polymer layer. Finally, CNFs were synthesized by heat treatment. Figure 24 summarizes the fabrication steps towards an array of CNFs with controlled geometrical structures using this approach. Firstly, the PAA is used as a mold (Figure 24a), then, nanoimprinting of the polymer was performed using a UV-photocurable monomer (Figure 24b), and finally, the nanofibers were formed applying a heat treatment at 1000 °C in vacuum for 3 h (Figure 24c). This fabrication process provides and straightforward methodology for the fabrication of a CNF array where the geometrical structures can be controlled by tailoring the porous structure.

**Figure 25:**
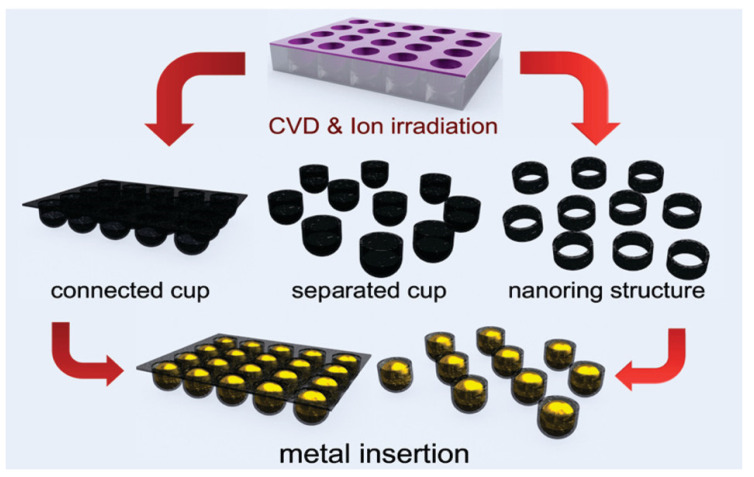
Fabrication approach to fabricate architectures of a connected arrays of nano-cup film, individually, separated nano-cups, nano-rings, and nano-containers for metal nanoparticles. Reprinted with permission from Ref. [225]. Copyright © 2023, the American Chemical Society.

In the work by Chun et al. [225] that adopted the template method, morphologies that differ from the typical tubular structures were explored obtained by tuning the PAA host matrix. Adjusting the anodization conditions, various carbon nanostructures were fabricated with precise nanoengineering of the length/diameter (L/D) aspect ratio of the porous alumina structure. Figure 25 summarizes the different carbon nanostructures obtained by adjusting geometry of the PAA templates. The approach consists of the implementation of an extremely short time (ranging from 20 s to 40 s) during the second anodization steps, then, a highly ordered PAA structure with short pores that leads to 10^3^–10^5^ times L/D aspect ratio is obtained. The synthesis of carbon nanostructures was carried out by the pyrolysis of acetylene at the temperature of 660 °C, without the use of any catalyst material. To obtain individual nano-architectures, Ar ion milling was applied on the connected arrays of a nano-cup film deposited in the PAA templates.

The graphitizing degrees of the synthetized nano-cup and nano-ring structures were analyzed by Raman spectroscopy with a red laser, in the spectral range 1200–1700 cm^−1^, where are typically the G band, ascribed to tangential modes of the graphene structure, and the disorder-induced D band, sensitive to defects in the graphitic structure [226]. The analyzed nano-cup and nanoring structures present a similar Raman spectrum as that of a MWCNT with a similar diameter. However, as the length is decreased (short nano-ring) the relative peak intensity I_D_/I_G_ is increased, indicating more degradation of the graphitic crystal structure as the L/D aspect ratio decreases. These nano-architectures have been filled with gold to probe their utilization as metal containers.

CNFs using metal NPs as a catalyst were synthesized by Che et al. [26] using an organometallic nickel solution that is immersed within PAA by diluting a nickel resinate toluene-based solution with different concentrations. To activate the catalytic activity of the NPs, different PAA/Ni composites were annealed under an inert atmosphere at 400 °C. Previously, it was mentioned that this method was adopted for the CNTs synthesis with an entangled nanotubes distribution emerging from the pores (see Section 3.1.2). In contrast, for CNFs, Che et al. [26] verified that individual solid CNF arises from each pore. 

**Figure 26:**
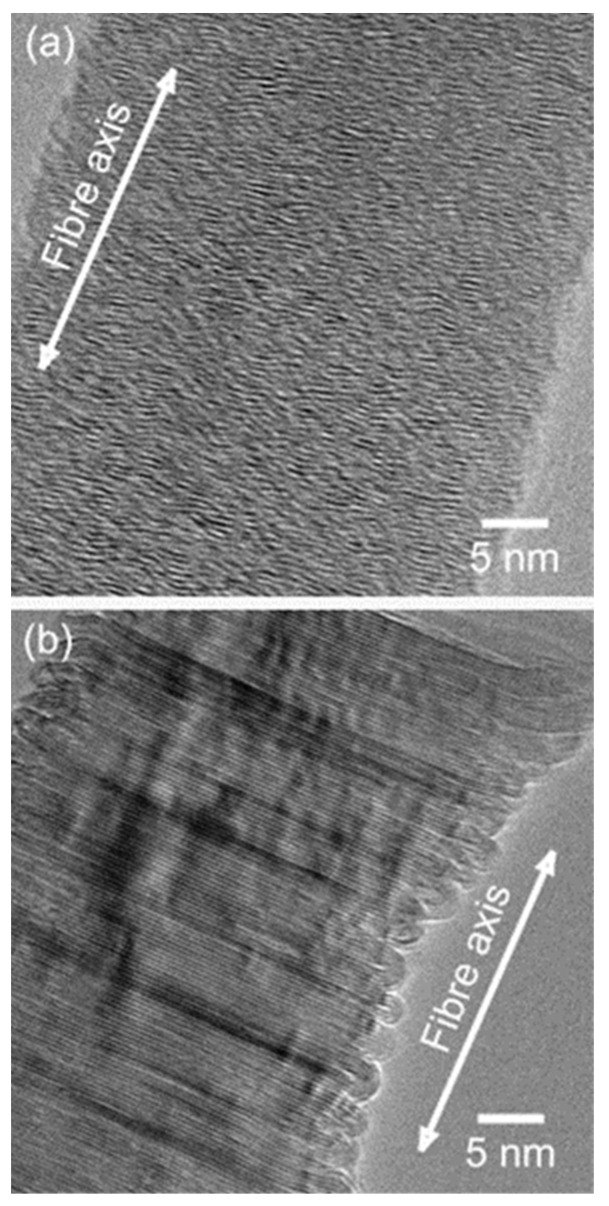
TEM images of the nanofilaments heated at: (**a**) 1000 °C and (**b**) 2800 °C. From the work carried out by Habazaki et al. [227]. Reprinted with permission from Ref. [227]. Copyright © 2023 Elsevier Ltd. All rights reserved.

Platelet structure carbon nanofilaments/nanofibers by liquid phase carbonization inside PAA templates have been studied for their implementation as an anode for power applications [227,228,229,230]. For the nanofilament fabrication, generally, polyvinyl chloride (PVC) powder was used as a carbon precursor. The PVC was immersed within the PAA and heated in inert gas at 300 °C, at this temperature, PVC decomposes to a liquefied pitch-like intermediate, and penetrates the PAA nanopores. Then, the composite is heated up to 600 °C for one hour. Finally, the template is dissolved in a wet etching 10% NaOH solution and the nanofilaments are filtered and subsequently heated in an inert atmosphere at temperatures ranging from 1000–2800 °C. Figure 26 shows TEM images of nanofilaments obtained by a heat treatment at 1000 °C and 2800 °C [227]. The platelet structure is present for both treatment temperatures. The fibers obtained at 1000 °C have the graphene layers normal to the fiber axis (Figure 26a), but at higher temperatures loops are developed at the edge of graphene layers where each adjacent 4–5 layers of graphene are connected by a loop (Figure 26b). The same kind of structure was obtained when the heat treatment was performed at 2400 °C [230]. 

**Figure 27:**
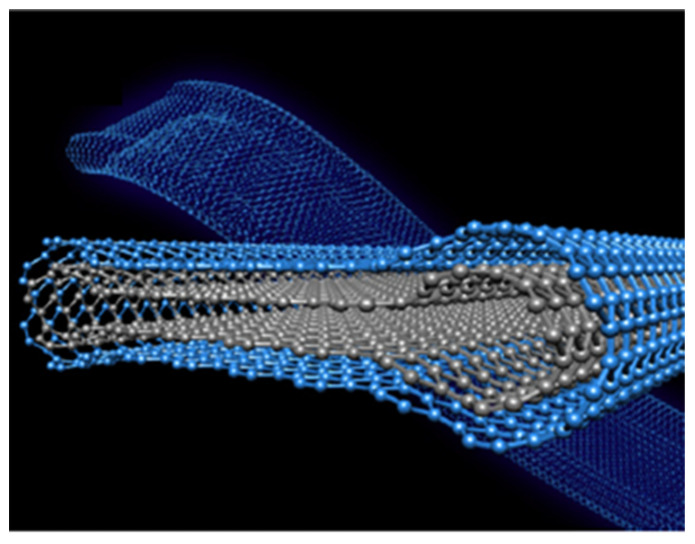
A scheme illustrating the structure of carbon nanobelt [231]. Reprinted with permission from Ref. [232]. Copyright © 2023 Elsevier Ltd. All rights reserved.

Another variety of carbon nanostructures synthetized inside PAA templates was developed by Lin et al. [231] using the template method and ethanol as a precursor; the authors coined this structure as carbon nano-belts. Figure 27 shows a schematic representation of the so-called carbon nanobelt obtained by the assisted template method. The formation mechanism of these nanobelts is similar to that proposed for CNTs. Initially, the carbon from the decomposed ethanol is coated on the pore walls surface and continuously grows as the carbon deposition evolves to form a thin film. Then, Lin et al. [231] proposed that the belt-like morphology is a result of the carbon layers collapse upon the removal of the template. As was previously mentioned, the template method for the synthesis of CNTs provides excellent control on the geometrical features of the carbon nanostructures but the crystallinity of as-synthesized products by Lin et al. [231] is relatively poor. Even if after annealing treatments it can be improved, the implementation for mostly electronic applications are severely limited.

**Figure 28:**
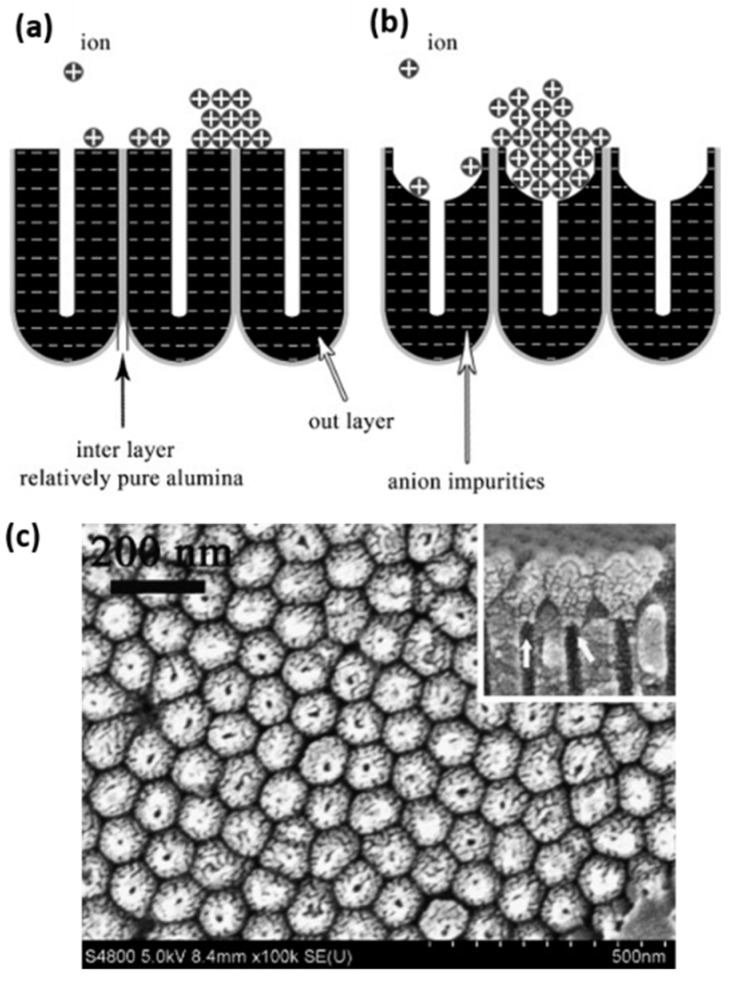
Schematic representation of the Carbon-nanodots nucleation and growth depending on the geometrical features of the PAA template (**a**) typical PAA template with straight pores, (**b**) modified PAA template with pore widening at the top surface, and (**c**) top view of nano-dot arrays after etching the PAA template. The inset depicts the cross-section view of nano-dots with small pits on the tips. Reprinted with permission from Ref. [233]. Copyright © 2023 Elsevier Ltd. All rights reserved.

Carbon nano-dots were synthesized through the filtered cathodic arc plasma (FCAP) technique [232,233,234]. The presented methodology includes a pore widening close to the PAA top surface, then, during the carbon deposition carbon ions are preferentially attracted by the localized inhomogeneous distribution of anion species [235] leading to nucleation and growth of carbon nano-dots in the widened pore instead through the whole surface, as schematically shown in Figure 28a,b. Therefore, by properly adjusting the pore widening, it is possible to obtain nano-dots with a hexagonal distribution (as shown in Figure 28c). This method could be interesting to be extended to other materials and explore different highly ordered nanotips arrays [236].

The most implemented methods for obtaining fullerenes C_60_ and C_70_ are based on vaporization of graphite by pyrolysis, radio-frequency-plasma, or arc discharge-plasma techniques [237]. These methods are not compatible with a selective growth of carbon nanostructures, in terms of their position and type of allotrope, consequently, purification and separation steps are required. Alternative chemical synthesis approaches have been proposed, but many challenges are remaining with these bottom-up approaches. For a deeper understanding of the state-of-art on the synthesis of C_60_ and C_70_ by chemical approaches, the reader can refer to the review article from Mojica et al. [238], where the problematics that face the synthesis of the variety of buckminsterfullerene are thoroughly described. Under this context, there is no reported synthesis of C_60_-C_70_ within PAA templates, but many researchers developed techniques to fill inside the pores under a direct current (DC) electric field and polymerized them [239,240] or by injecting C_60_ based solutions inside the PAA templates known as the diaphragm liquid-liquid interfacial precipitation (DLLIP) method [241,242]. 

In summary, a wide diversity of fullerenes has been synthesized or integrated within PAA templates. The presented synthesized carbon nanomaterials are representative of smart utilization of the PAA templates for the nanofabrication of PAA/Carbon-based assemblies. Currently, the implementation of these materials is mainly used in energy conversion and storage devices [243].

## 4. Applications Based on PAA-Carbon Nanostructures

**Figure 29:**
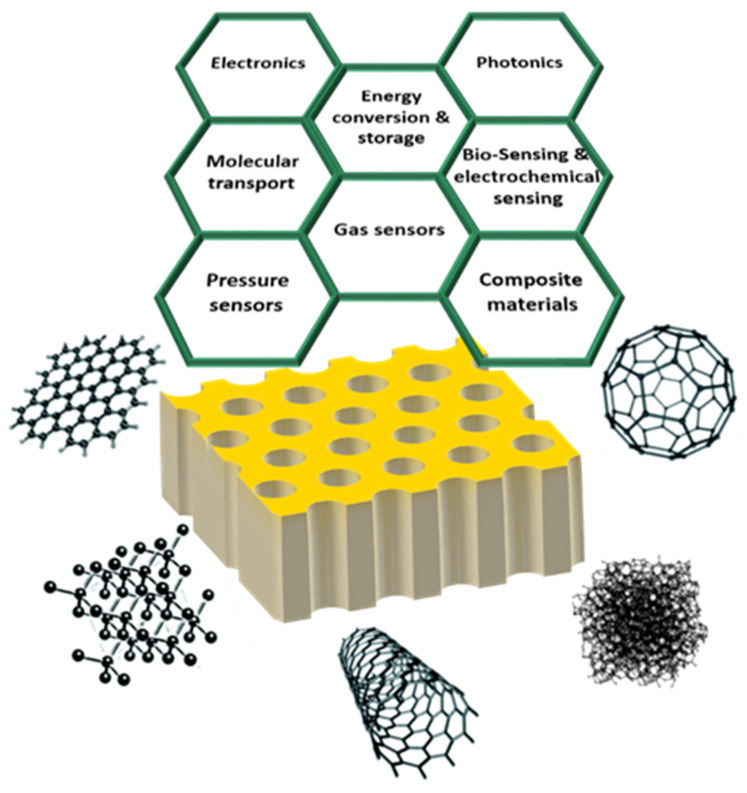
Various applications developed over the years based on PAA/CNTs assemblies.

A wide diversity of applications based on PAA-carbon nanostructures have been investigated during the last past decades taking advantage of the versatility the PAA offers to adjust the geometrical features and the chemical compatibility with the synthesis of carbon nanostructures or by simply transferring them to the template. Figure 29 summarizes the multiplicity of applications based on carbon nanostructures grown inside the PAA template or transferred to a template such as graphene or C_60_. In the present review, we highlight many representative applications in a wide range of fields.

### 4.1. Electronic Devices Based on PAA/CNTs

The pursuit of innovative semiconducting materials to develop nanoelectronics devices is a topic that requires tremendous efforts to continue with the device shirking to extend Moore’s law. SWCNTs are very promising and extensively investigated for electronics due to their high carrier mobility and one-dimensional scalability [244].

**Figure 30:**
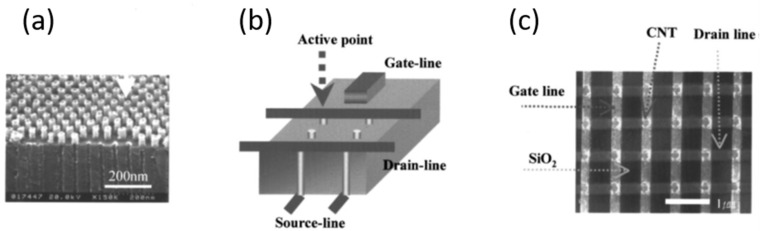
Transistors based on PAA/CNTs material. (**a**) Device architecture with individual unit devices consisting of CNT, (**b**) which is at the cross point of bottom and (**c**) top electrode the SEM cross-section and top views show the CNT array. Reprinted with permission from Ref. [245] Copyright © 2023, AIP Publishing.

Transistors with a vertical geometry have been fabricated growing CNTs inside PAA templates. Figure 30 summarizes two emblematic works that fabricated transistors based on PAA/CNT. Both studies are interesting from a proof-of-concept perspective. Choi et al. [245] took advantage of the versatility of the PAA template that enables the engineering of individual devices with a device density in the tera level per cm^2^ (2 × 10^11^/cm^2^). It consists of vertical carbon nanotubes attached, respectively, to a bottom source and an upper drain electrode and an electrostatically switchable gate electrode. Figure 30a–c summarize SEM images of a cross-section and top view of the vertically aligned CNTs grown in the patterned PAA and the schematic device architecture of the CNT transistor. The downside of vertical carbon transistors is the low working temperature (30 K). This is attributed to the MWCNTs implementation as the active semi-conductive element which is semi-metallic at room temperature.

Alternatively, Franklin et al. [246] proposed an approach to fabricate SWCNT/PAA transistors. As described in Section 3.1.2, SWCNTs can be grown inside PAA templates using an Al-Fe-Al structure as starting material for the Al anodization and further nanostructure synthesis. CNTFETs were fabricated from SWCNTs grown from the PAA and contacted with Pd source/drain contacts. Interestingly, the contact between the SWCNT and the metal electrode takes place during the CVD process. However, this methodology leads to a drastic reduction of the device density. To sort semi-conductive and metallic tubes, a burning process was required where metallic ones are removed [247]. It’s worth noting that for an easy testing procedure, the density of SWCNTs in the template was kept extremely low by performing synthesis for times of 1 min or less.

The previous cases of electronics applications based on PAA/CNTs assemblies were reported in proof-of-concept perspective. Even if the PAA templates offers a nice host for 1D carbon nanostructures, the ordering and orientation of the tubes are lost when the tubes emerge from the pores. On the other hand, the template-based method guarantees the collectively order of the MWCNTs, but these have high density of structural defects leading to tubes with poor electronic transport properties. Then, this kind of application are severely limit since seems very complicate to conciliate the collectively ordering and the low structural defects amounts of the carbon nanostructures synthetized inside the PAA templates.

### 4.2. Energy Conversion and Storage Devices Based on PAA/Carbon Nanostructures Composites

**Figure 31:**
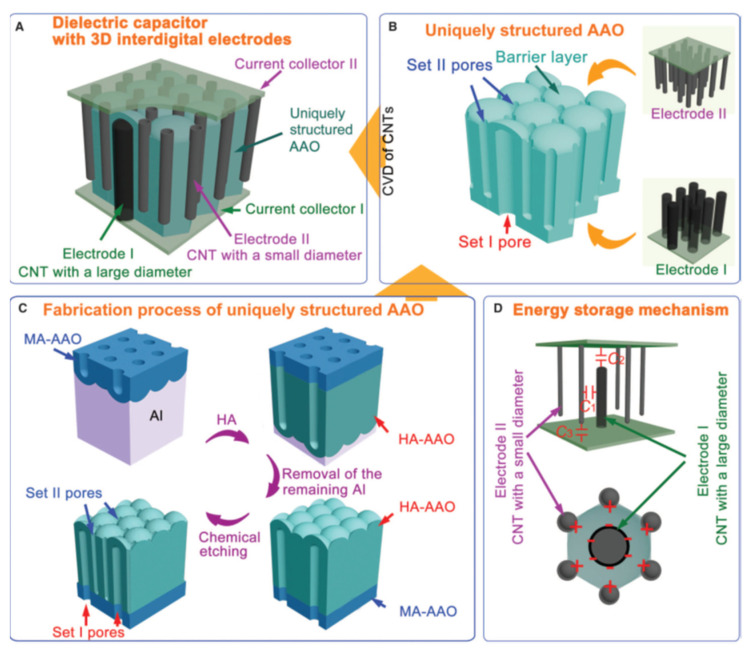
Schematic depiction of the structure, fabrication process, and energy storage mechanism of the designed dielectric capacitor. (**A**) Dielectric capacitor with a 3D interdigital electrode. (**B**) Breakdown structure of the dielectric capacitor. (**C**) The fabrication process of the uniquely structured PAA membrane. (**D**) Schematic depiction of the energy storage mechanism of a unit cell in the newly structured dielectric capacitor from side view (top) and top view (bottom). Reprinted with permission from Ref. [253]. Copyright © 2023, AAAS.

Highly dense-packed nanostructured arrays are of great interest for the next generation of energy storage devices. Recently, a very interesting and exhaustive review paper was reported by Kim et al. [248] summarizing the synthesis and processing of a variety of carbon nanostructures and demonstrations of their potential applications such as flexible and transparent super-capacitors [249], high-performance supercapacitors [250], and nano-containers [251]. The review article highlights that energy conversion and storage devices, thanks to the PAA versatility to precisely tailor low aspect-ratio structures compatible with carbon nano-architectures using a CVD process due to their excellent features such as high transparency, flexibility, regularity, and large specific surface area. Particularly, dielectric capacitors are very attractive due to their high-power density and increasing energy density. The rapid development of renewable energy technologies is highly dependent on the performance of energy storage devices since they still require enormous effort to improve their energy density and storage efficiency. Then, nanomaterials are of great interest due to the high specific surface that they can provide. For instance, the most implemented dielectric capacitors are basically made from two metal electrodes separated by a solid dielectric film [252]. Han et al. [253] proposed a 3D nano-architecture design using a smart tuning of PAA templates, where two sets of interdigitated and isolated straight pores are obtained by the application of MA and HA processes. Both sets of pores are opening toward opposite planar surfaces and, subsequently, two sets of CNTs were grown by CVD inside both kinds of pore’s type. A catalyst-free approach was adopted to fabricated the PAA/CNT structure. Finally, the dielectric capacitor was obtained after gold films were sputtered onto the two planar surface sides of the PAA/CNT structure. Figure 31 summarizes the geometrical and structural features, fabrication process, and energy storage mechanism of the designed dielectric capacitor arrays, using the PAA as a dielectric layer with two sets of pores sizes used as plates by embedding them with CNTs. The arrangement of interdigital electrodes of this unique PAA/CNT cell structure is based on large-diameter CNT surrounded by six small-diameter CNTs. In regard to the energy storage device performance, the presented PAA/CNT device present a capacitance density of about 47 mF/cm^2^ when a 6-mm-thick HA-PAA was used. The breakdown voltage was about 15 V. The dielectric capacitor can reach an energy density of about 2 Wh/kg. The fabricated dielectric capacitor compared with the reported data in the literature has a superior performance in terms of energy density with respect other metal-insulator-metal capacitors built in porous materials. This kind of hybrid nanodevice has great potential to pave the way for the next generation of energy storage device, and once again the versatility of PAA as a template is reflected [254].

**Figure 32:**
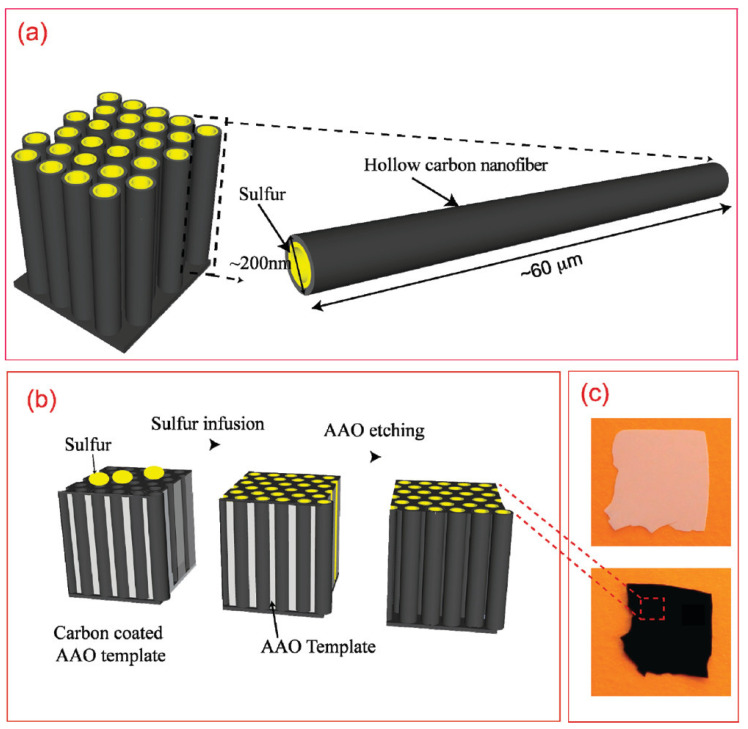
Schematic design and fabrication process of hollow CNFs/sulfur composite structure. (**a**) The design principle shows the high aspect ratio of the hollow carbon nanofiber for effective trapping of polysulfides and (**b**) the fabrication process of carbon/sulfur cathode structure. (**c**) Digital camera images showing the contrast of the PAA template before and after carbon coating and sulfur infusion. Reprinted with permission from Ref. [255]. Copyright © 2023, the American Chemical Society.

Regarding lithium-ion applications, hollowed CNFs have been synthesized inside PAA templates and further sulphur encapsulation has been performed to develop cathodes for high specific capacity rechargeable lithium-ion batteries [255]. Once again, the catalyst-free approach to synthesized the carbon nanostructure is useful to grow the CNF with a control diameter and open caps in both extremes. Figure 32 shows the geometrical features of the encapsulated hollowed carbon nanofiber with sulphur and their workflow to develop a carbon/sulphur cathode structure. The carbon coating of the PAA was achieved through a polystyrene carbonization process. Then, the sulphurisation process took place with a controlled amount of 1% sulphur solution in toluene and to ensure the sulphur diffusion into the pores an annealing step was executed. The fabricated cathodes developed by Zheng et al. [255] enable high-performance Li-S batteries with a specific capacity of about 730 mAh/g after 150 cycles of charge/discharge at C/5 (C/n refers to the battery discharge rate, C/5 implies a five-hour discharge battery), revealing the outstanding electrochemical performance of the hollow carbon nanofiber-encapsulated sulphur cathodes [256]. In a subsequent study [257], the hollow CNFs was modified with an amphiphilic surface to improve the cycling performance. This strategy was adopted to stabilize the subproducts generated during the discharge. Consequently, the capacity of the sulfur-based cathodes was increased up to 1180 mAh g^−1^ at 0.2 C and maintained an 80% storage capacity after 300 cycles at 0.5 C. Lithium-sulfur batteries have a theoretical capacity of 1672 mAh g^–1^ [258], indicating the good performance established by the PAA/CNF/sulphur-based batteries developed by Cui group. However, there are still various challenges remaining to develop applications based on Li-S batteries with a competitive energy density compared with commercial Li-ion batteries [259,260].

Electrocatalysis is another foundation stone for the development of renewable energy technologies. Currently, most of the high-efficiency oxygen reduction reaction (ORR) and oxygen evolution reaction (OER) are depending on IrO_2_/RuO_2_ and Pt-based materials. Nevertheless, practical, and scalable applications based on this group of materials are severely threatened by the high cost and their relatively low abundance on earth. To address this issue, various hybrid materials have been proposed to achieve cost-effective alternatives. Particularly, CNTs are very attractive due to their already previously mentioned physical/chemical properties and their straightforward methods for functionalization. There are in the literature interesting reviews that present a complete overview of the recent progress in CNTs applied on ORR/OER [261,262].

**Figure 33:**
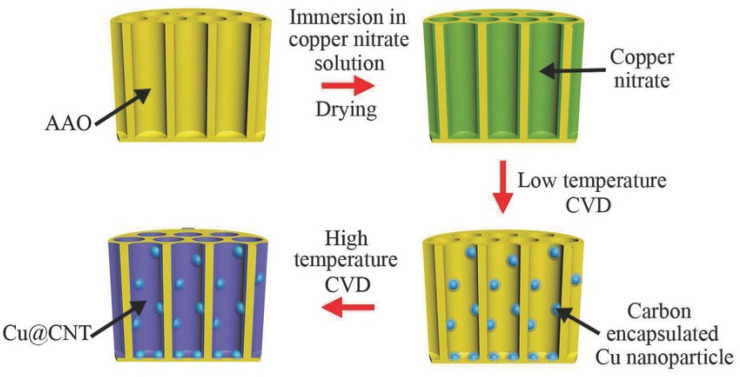
Schematic illustration of the procedure implemented by Zhao et al. [263] of Cu NPs embedded CNTs grown on PAA templates. Reprinted with permission from Ref. [263]. Copyright © 2023 WILEY-VCH Verlag GmbH & Co. kGaA, Weinheim, Germany.

Zhao et al. [263] took advantage of the PAA template properties to load the pore walls with copper nitrate, followed by a low-temperature CVD process to reduce Cu NPs under acetylene pyrolysis at 500 °C. Then a CVD step at high temperature at 800°C was performed to form the CNTs. Figure 33 shows a schematic representation of the fabrication process for the formation of Cu NPs embedded CNTs, named Cu@CNTs. Interestingly, the authors also exanimated the ORR activity of pure CNTs and a variety of hybrid CNTs materials obtained from the Cu@CNT composite. For instance, Cu NPs embedded in Nitrogen-doped material, named Cu@NCNTs were fabricated. Doped nitrogen atoms and Cu–N species promote additional active sites for ORR. 

Similarly, previous works prove that cobalt oxide nanohybrids at the interface between cobalt oxide and carbon nanomaterials showed high ORR current densities [264], then, CoxOy nanoparticles were decorated on the Cu@NCNT surfaces, calling the composite Cu@NCNT/CoxOy. As a result, the synergistic coupling between CoxOy nanoparticles and Cu@NCNTs leads to a non-precious metal-based composite with a high-performance ORR catalytic activity. From a material fabrication perspective, the proposed preparation also entails an innovative approach to decorate CNTs thanks to the PAA properties. Consequently, this approach can be extended to a wide variety of hybrid materials obtained from the PAA/CNT nanocomposite. All the experimental procedures for the composites fabrication are given in detail in the work performed by Zhao et al. [263].

Highly durable platelet-type CNFs (pCNFs) based electrodes for OER/ORR in alkaline media have been developed by Habazaki groups. In a first approach, Tsuji et al. [229] prepared pCNFs by liquid-phase carbonization inside the PAA template as was previously described in Section 3.4. Different thermal treatments were applied to reduce the graphene edge sites that play a key role during the ORR [265]. These edges can be easily controlled by thermal treatments. Subsequently, the carbon nanostructures were further functionalized with Pt NPs. This hybrid material shows an improvement of ORR activity and the durability of the platinum increased with the heat-treatment temperature. In particular, the electrochemically active surface area and ORR activity of the Pt NPs deposited on the CNFs heat-treated at 1400 °C maintained more than 90% of their initial value after potential cycling from 0.5 to 1.5 V vs. RHE for 200 cycles. The authors attributed this performance to the fact that edge atoms exposed at the side walls of pCNFs thermally treated at high temperatures result in strong stabilizing interaction with the Pt NPs.

More recently, Sato et al. [230] similarly, prepare the pCNFs inside the PAA template by the liquid-phase carbonization method. Interestingly, the authors analyzed the OER/ORR in alkaline media using different carbon nanostructures. For instance, MWCNTs, (DB2400) is a stack of particles with graphene layers randomly distributed, pCNF1500 and pCNF2400, which refers to structures with graphene layers arranged perpendicularly to the fibre axis of the nanofiber synthesized at 1500 °C and 2400 °C, respectively. In these cases, all the fabricated electrodes were precious metal-free. Interestingly, the authors found a dependence between the graphitic orientation with respect to the nanostructure axis and the OER activity. For instance, among the studied materials, the pCNFs are the most durable under OER conditions without significant decay of the electrocatalytic activity after one month of testing. On the contrary, the exposure of basal planes with the alkaline electrolytes led to a change in the carbon degradation process from general-type to pitting-type corrosion associated with faster oxidation kinetics. The results in this study reveal that the high oxidation resistance of carbon edge planes in highly graphitized CNFs can lead to a new design of durable air electrodes than using just carbon materials as conductive support.

### 4.3. Molecular Transport within PAA/Carbon Nanostructures

**Figure 34:**
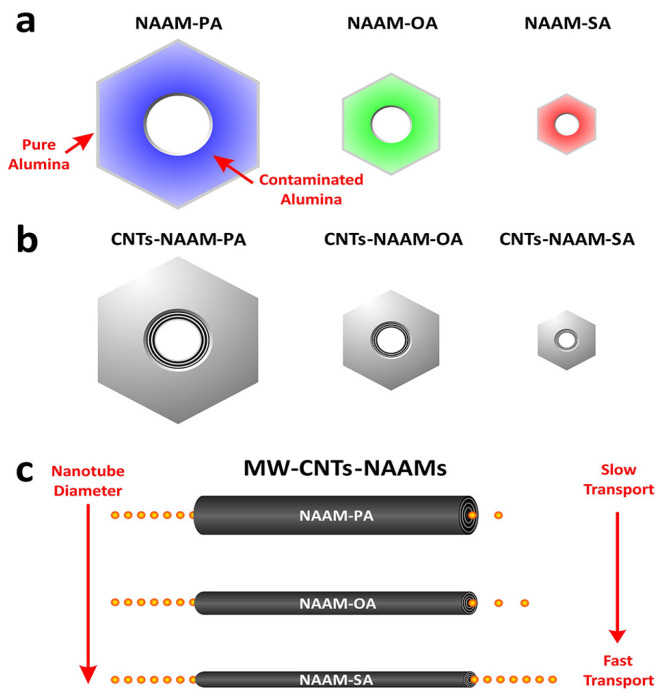
Schematic representation of the chemical composition of the fabricate PAA templates executed by Alsawat et al. [28]. (**a**) Different anion distributions on the Chemical composition of PAA templates are obtained using different electrolyte solutions, where PA, OA and SA refer to Phosphoric Acid, Oxalic Acid and Sulfuric Acid, respectively. (**b**) Fabricated PAA/CNTs assemblies after the CVD process, and (**c**) transport through PAA/CNTs assemblies depending on the geometrical PAA feature. Reprinted with permission from Ref. [28]. Copyright © 2023 the American Chemical Society.

The fabrication of CNT composite membranes with controllable dimensions (pore diameters and length) enables to target applications related to molecular transport [28,145,266,267], filtration [268] and surface wettability [268,269]. Losic group studied in detail the transport performance of the PAA/CNTs composite using various dye molecules (Rose Bengal, (RosB)^2−^, (Ru(BPY)_3_)^3+^ and RhoB). The authors concluded that the transport properties are mainly determined by the geometrical features of the pore walls composites as depicted in Figure 34. Then, the influence of the anionic distribution in the pore walls of the fabricated PAA has a negligible influence. For instance, it was verified that PAA anodized with phosphoric acid solution leads to a membrane PAA/CNTs with a hydrophilic character than those fabricated using other electrolytes (i.e Oxalic acid and Sulphuric acid) but no enhancement on the transport of the hydrophilic dye molecules was obtained. 

An interesting study was carried out by Mattia et al. [268], where the authors verify that the permeability is dictated by the solid-liquid molecular interactions between the liquid and the CNTs structure. Besides, the authors perform an exhaustive overview pointing out the transport properties of PAA/CNTs membranes in terms of selectivity and permeability in comparison with the commercial polymer-based membranes, revealing that the PAA/CNTs composites have a higher pure water permeability than the available commercial ones. 

The fabricated PAA/CNTs membranes can also be used as a filter for specific molecules, Jafari et al. [270] evaluated the transport properties of Humic acid (HA). The membrane rapidly absorbs HA and consequently, a rapid flux decay takes place due to the internal pore constriction as the dominant fouling mechanism. On the other hand, the authors point out that despite the superior pure water transport performance of the PAA/CNTs membranes, the fragility of the PAA/CNTs membrane can compromise their implementation as a filter pollutants removal from water in large surface or high-pressure applications.

The ionic transport using an assembly of PAA/Graphene was studied by Akhtar et al. [206], introducing 0.5 M potassium chloride (KCl) and deionized water in a side-by-side Franz diffusion cell. It was found that the samples with graphene sheets on top of the PAA template with a 20 nm average pore diameter block 66% ions transport. The presented results are quite promising to develop PAA/graphene membranes with potential applications in water desalination and gas purification applications after further PAA optimization.

### 4.4. Photonic Devices Based on Cup-Stacked CNTs Grown inside Lateral-PAA Templates

**Figure 35:**
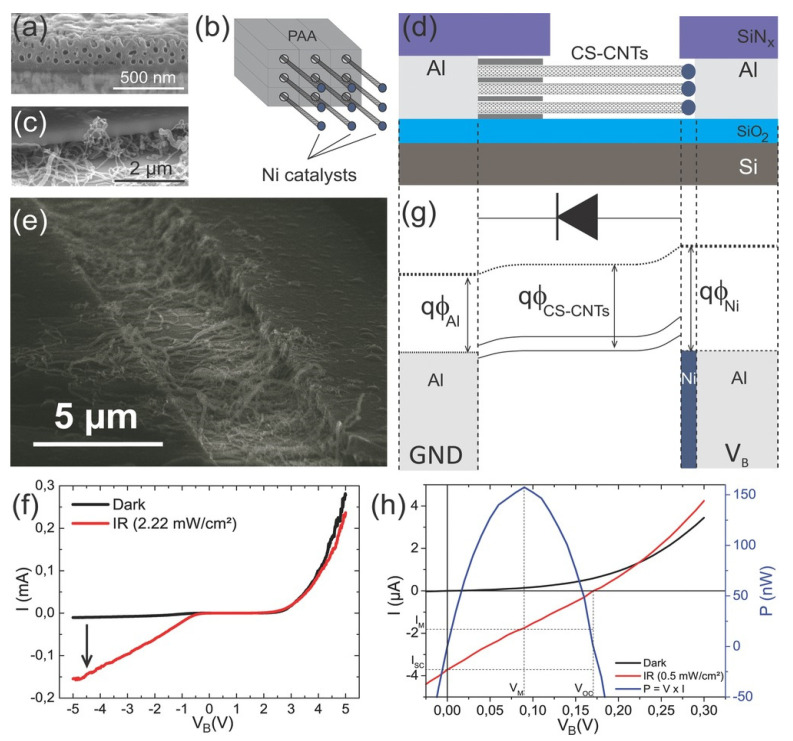
Schematized the geometry of both adopted approaches to engineering Schottky diode devices based on cup-stacked carbon nanotubes arrays grown in lateral-PAA anodized alumina templates asymmetrically contacted. (**a**) SEM image of lateral-PAA without capping layer before the CS-CNTs, (**b**) ketch of CS-CNTs emerging from the pores, (**c**) SEM image of Lateral-PAA with SiNx capping layer after CS-CNTs growth. (**d**) Sketch of CS-CNT Schottky diode. (**e**) SEM image of a channel between the lateral-PAA and aluminum electrode contacted by CS-CNTs. (**f**) Current-voltage curve of the photo-diode device in the dark and under iR illumination. (**g**) Band-diagram of the lateral-PAA/Cs-CNTs device at zero bias. (**h**) Magnified current, bias voltage, and power characteristics around the origin in the dark (black line) and under illumination (red line). Reprinted with permission from Ref. [14]. Copyright © 2023 WILEY-VCH Verlag GmbH & Co. KGaA, Weinheim, Germany.

The presented applications benefit from the collective organization that the PAA templates offer in the orthogonal direction of the substrate. However, horizontally aligned CNTs arrays directly synthesized in substrates are attractive for multiple applications [271,272]. Lateral-PAA enables the organized growth of nanostructure in the direction parallel to the substrate. Optoelectronic devices based on cup-stacked carbon nanotubes (CS-CNTs) grown inside Lateral-PAA templates have been developed for photovoltaic and photodetectors applications by Kim et al. [14]. Two different approaches have been adopted to create an asymmetrical contacted array of nanotubes leading to Schottky photodiodes. The first approach relies on the contact between the catalyst NP from the carbon nanostructure to an aluminium metal electrode. Then, the formation of the Schottky contact takes place during the CVD process. Figure 35 summarizes the Lateral-PAA/Cs-CNTs photonic devices, depicting the device geometry and performance.

**Figure 36:**
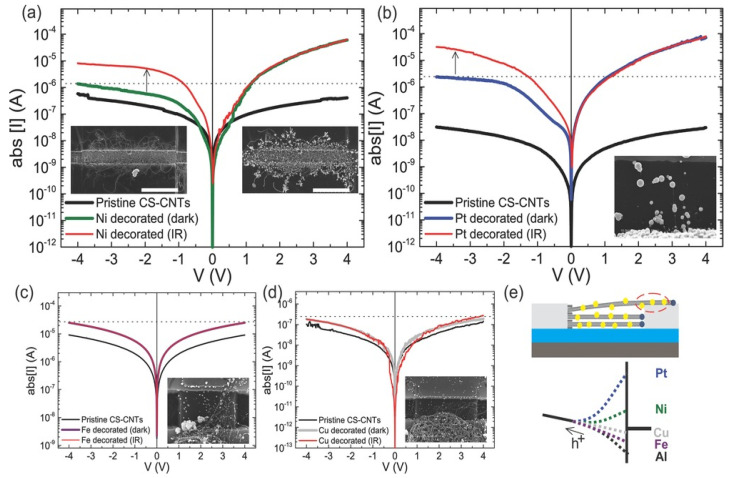
Lateral-PAA anodized alumina templates asymmetrically contacted obtained via an electrodeposition process. A dependence between the values of the corresponding metal NP work functions and the rectifying effect has been established in a Schottky Barrier model. (**a**) I–V curve characteristics of a CS-CNTs device before Ni decoration in the dark (black line), after Ni decoration in the dark (green line) and under IR illumination (red line). Left inset: SEM image before decoration. Right inset: SEM image after decoration. Scale bar: 20 μm. (**b**) I–V characteristics of a CS-CNTs device before Pt decoration in the dark (black line), after Pt decoration in the dark (purple line) and under IR illumination (red line). Right inset: SEM image after decoration. Image size: 10 μm × 5 μm. (**c**) I–V characteristics of a CS-CNTs device before Fe decoration in the dark (black line), after Fe decoration in the dark (lilac line) and under IR illumination (red line). Right inset: SEM image after decoration. Image size: 20 μm × 10 μm. (**d**) I–V characteristics of a CS-CNTs device before Cu decoration in the dark (black line), after Cu decoration in the dark (grey line) and under IR illumination (red line). (**e**) Top: a sketch of a metal decorated device. The dashed ellipse shows the NPs responsible for the Schottky Barrier tuning. Down: a sketch of the corresponding energy band diagram for the different metal nanoparticles showing the tuning of the Schottky Barrier at the contact. Reprinted with permission from Ref. [14]. Copyright © 2023 WILEy-VCH Verlag GmbH & Co. KGaA, Weinheim, Germany.

Alternatively, similar performances were obtained through a surface modification of the CS-CNTs via an electrodeposition process. In this approach, the CS-CNTs surface acts as preferential sites for the NPs nucleation during the electrodeposition process. Then, the metallic nanoparticles decoration can break the symmetrical contact, transforming CS-CNTs resistors into Schottky diodes. Interestingly, the electrodeposition process allows the depositing of various types of NPs with different work functions. As shown in Figure 36, the work function of the decorate NP plays a critical role to tailor the Shottky diode. Even if the asymmetrical contact is achieved through the surface modification, the following relationships among the work functions values of each element must be preserved: $\varphi_{Al}<\varphi_{CS-CNT}<\varphi_{Metal\;decorated}$, where $\varphi_{Al}$ refers to aluminium metal electrode work function, $\varphi_{CS-CNT}$ denotes the CS-CNTs and metal NP work functions. 

This kind of device is very attractive because a large surface of the nanostructure is exposed to the environment making them suitable for many sensing applications. On the other hand, the difficulty to control the carbon nanostructure growth evolution after the 1D material emerges from the pores remains the main challenge of these kinds of devices.

### 4.5. Gas Sensors Based on PAA/CNTs Devices

**Figure 37:**
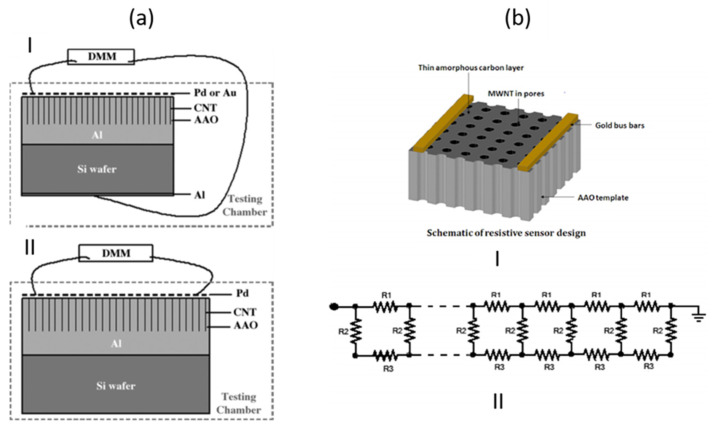
The different metal electrodes configurations were proposed to develop PAA/CNTs gas sensors. (**a**) Two different metal electrodes configuration have been proposed by Ding et al. [274], metal electrodes were deposited on the top PAA surface and on the Si wafer backside where the PAA was anodized. Reprinted with permission from Ref. [274]. Copyright © 2023 Elsevier Ltd. All rights reserved. (**b**) Two gold bars have been deposited as metal electrodes on the top surface, then, the amorphous carbon layer on the top surface electrically connects the CNTs grown inside the pores [282]. Reprinted and modified with permission from Ref. [282]. IOP Science Copyright © 2023.

Gas sensors based on CNTs synthetized within PAA templates have been also explored by some authors [273,274,275,276]. The fabricated resistive gas sensors devices showed a moderate response to NH_3_, NO_2_ and H_2_. Figure 37 summarizes the different chemiresistive device designs based on PAA/CNTs. Regarding gas sensing application, the PAA structure dramatically decreases the gas molecule interaction of the CNTs with the gas molecules when the metal electrodes are deposited on the top and bottom PAA surfaces, as in Figure 37a (I), as proposed by Ding et al. [274], no detectable changes in the electrical properties are detected when the device was exposed to H_2_ environment. Previous works reported a change in their electrical resistance when H_2_ in the gas phase are exposed to pristine MWCNTs [277]. Then, the absence of signal variation, in this metal electrode configuration, is attributed to the absence of the CNTs surface exposure to the environment. In the second configuration proposed, Figure 37a (II), the system reacts to the environment since the Pd film that supports mainly contributes to the total electrical transport, consequently, the Pd film is the main active element of this device.

Alternatively, in Rajaputra et al. [275], as shown in Figure 37b (I) two metal electrodes also have been deposited on the top surface but in this case, the amorphous carbon deposited during the CVD process on the top and bottom surface plays a critical role, electrically connecting the individual CNT grown inside the pores. Otherwise, the nanotubes are isolated from each other resulting in an open circuit due to the dielectric PAA behaviour. Mangu et al. [276], as shown in Figure 37b (II) proposed and developed an equivalent resistance model that takes into consideration the resistance of the amorphous carbon on the top and bottom PAA surfaces between two adjacent MWCNT’s. The authors show that this kind of device presents a cumulative sensor response at a concentration of 0.01% NH_3_ of 15.37% up to 17.7% depending on the amorphous carbon layer thickness. The device response for a chemiresistor is conventionally defined by $R=\left(\frac{I\;-\;I_{0}}{I_{0}}\right)\times 100\%$, where I is the device current density under the target gas and $I_{0}$ the current density under a carrier gas (typically dry air or an inert gas) [278].

Capacitive gas sensors have been also developed by Chen et al. [279]. Since two metal electrodes were evaporated on both sides of the PAA surfaces to fabricate the capacitive device, a chemical etching was performed using 10% HF at room temperature for 300 min to expose the CNTs to the environment. Similarly to the chemiresistive devices, the gas sensor response ($R_{C}$) to a target gas was defined by $R_{C}=\left(\frac{C\;-\;C_{0}}{C_{0}}\right)\times 100\%$, where C is the capacitance of the capacitor exposed to the target gas and where $C_{0}$ is the base line capacitance. With this device much lower NH_3_ concentrations were detected, showing a 2% response when the device was exposed to 1 ppm of NH_3_. Although, these PAA/CNTs based gas sensors have moderate responses compare to other gas sensors based on nanomaterials [280,281]. The proposed configuration is mainly attractive because the fabrication process is straightforward and they do not involve complex fabrication techniques like e-beam lithography or photolithography. Furthermore, the chemical stability of the PAA/CNTs make this composite material suitable to operate in harsh environments.

### 4.6. Biosensing and Electrochemical Sensing

During the last decades, many efforts have been devoted to developing biosensing platforms of relatively low cost that can fast and accurately detect and quantify biomarkers associated with many conditions or diseases [131]. Biosensors are devices that transduce a bio-chemical event into a signal (electrical or optical) [283,284]. The biocompatibility of carbon nanomaterials has developed the field of electrochemical sensing of a wide range of analytes. For instance, the diagnosis and management of diabetic patients need accurate monitoring and control of the glucose level in the blood. To this purpose, Claussen et al. [285] fabricated networks of SWCNTs with electrodeposited Au-coated Pd (Au/Pd) nanocubes to develop electrochemical biosensors to amperometrically sense hydrogen peroxide (H_2_O_2_). The fabricated device exhibited high sensitivity toward H_2_O_2_ guaranteeing excellent oxidase-based biosensing.

**Figure 38:**
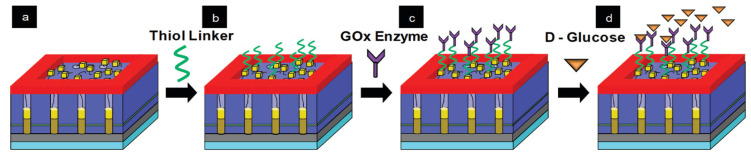
Schematic representation of the electrode bio-conjugation process steps to develop the glucose biosensor. (**a**) SWCNTs grown from the pores of the PAA via MPCVD with decorated Pd/Pt (Pd to form Pd nanowires in pores and Pd nano-cubes on SWCNTs), further electrodeposition is also implemented to coat the Pd or Pt nano-objects. Al2O3 is patterned to define electrode surface area. (**b**) thiol covalent linking of dithiobis (succinimidyl undecanoate) to Au/Pd nano-cubes or Au/Pt nanospheres. (**c**) covalent linking of GOx enzyme to thiol linker, and (**d**) attachment of d-glucose molecules to selective GOx sites. Reprinted with permission from Ref. [285]. Copyright © 2023 the American Chemical Society.

Further two-step biofunctionalization was performed to engineer a glucose biosensor, based on a thiol covalent linking of dithiobis (succinimidyl undecanoate) to Au/Pd nanocubes and covalent linking of glucose oxidase (GOx) enzyme to a thiol linker as illustrated in Figure 38. The implemented procedure is very advantageous since in situ decoration of Au/Pd nanocubes on as-grown SWCNTs eradicates complicated sorting, washing, and post-processing methods involved in most SWCNT-electrode immobilization schemes. The electrode was demonstrated as a glucose biosensor by immobilizing GOx on the surface of the nanocubes via thiol linking. As a result of the GOx biofunctionalization, the amperometric glucose sensing is improved in terms of the glucose detection limit, linear range, and response time compared to the biosensors device based on the PAA/SWCNTs decorated device.

Taking advantage of the merits of this biosensing platform, such as the high sensitivity toward H_2_O_2_ the biocompatibility of Au/Pd nanocubes, in another work, Claussen et al. [286] similarly developed Pd-nanocubes and Pt-nanospheres electrodeposited on SWCNTs networks grown inside PAA templates, after immobilizing the enzyme glutamate oxidase (cross-linked with glutaraldehyde) onto the electrode surface the device was made capable to be sensitive to glutamate molecules. This is very interesting for neurological related conditions since it is the major excitatory neurotransmitter in the nervous system [287]. The authors found that Pt nanosphere/SWCNT biosensor outperformed the Pd nanocube/SWCNT biosensor with a wide linear detection range and low limit of detection, resulting in one of the highest performing enzymatic electrochemical sensors based on nanomaterials [288].

An innovative immunoassay sensing platform has been recently developed by Qiao et al. [289], based on pores walls of commercial PAA membranes and the ion pair interaction mediated by electrochemistry of C_60_. This study brings together the PAA membrane-based label-free biosensing [290] and redox activity of C_60_ [291]. Inspired by a previous work, where the changes in the steric hindrance were monitored using Fe(CN)_6_]^3–^ as a reference [292], the PAA nanochannels were the matrix to immobilize the antibodies that in presence of the target protein leads to the formation of immunocomplex variation of the steric hindrance of the nanochannels. The authors concluded that this kind of electrochemical device based on redox indicator is very interesting to develop a large variety of nanochannel-based bio-arrays.

Regarding electrochemical sensing applications, Kim et al. [293] developed an ion-sensitive field-effect transistor (IS-FET) sensor with the capability to directly detect nitrate in water without any buffer solution. This kind of device can offer an alternative to the current technologies based on colorimetric and ion-selective electrode (ISE) techniques [294]. However, both methods have their limitations, for instance, with the colorimetric based technology, the detection can determine elements in water with concentrations ranging from 1–15 ppm which is useful for rough estimations but not for accurate monitoring [295]. The ISE based devices are sensitive and rapid sensors, but the sensor probe is very fragile and difficult to handle demanding a high maintenance cost. Then, the IS-FET device based on a free-standing PAA support for the graphene and Au electrodes has a great potential in the field of water quality analysis since the authors reported a limit of detection of 0.05 ppm with a response time lower than 3 seconds. The outstanding sensor performance is attributed to the shift of the Dirac point of graphene after coating the selective membrane. Consequently, the performance of the device is significantly enhanced due to the porous structures of the PAA.

### 4.7. Pressure Sensor Based on Transferred Wrinkled Graphene

**Figure 39:**
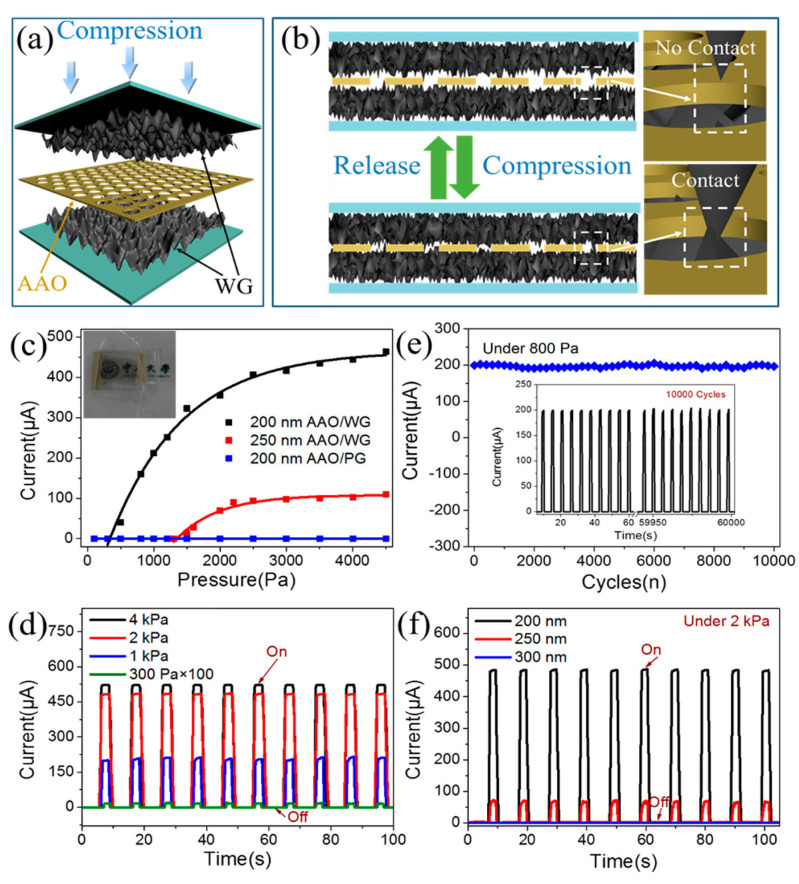
Schematics illustration of the PAA/Graphene device and current responses of pressure sensors. (**a**) Structural and (**b**) operating schematics of the pressure sensor. (**c**) Current response of different pressure sensors under various pressures. Inset is the photograph of packaged pressure sensor with silver electrodes. (**d**) Cyclic current responses of pressure sensors based on 200-nm-thick AAO membrane and WG under various pressures. The cyclic current response of the pressure sensor under 300€ was multiplied by 100. (**e**) Stability measurement of pressure sensors based on 200-nm-thick AAO membrane and WG. (**f**) Cyclic current responses of pressure sensors based on PAA membrane with different thickness and WG. Reprinted with permission from Ref. [208]. Copyright © 2023 WILEY-VCH Verlag GmbH & Co. KGaA, Weinheim, Germany.

Considering applications based on a PAA/Graphene assembly, Chen et al. [208] have developed a high sensitivity, ultrathin, and transparent pressure sensor using pristine graphene (PG) and wrinkled graphene (WG). This kind of mechanical pressure sensor is very promising for the development of wearable electronics and devices integrated for IoT. The device configuration also can be used as an electrical switch when the pressure sensor is under compression. The pressure sensor based on WG/200-nm-thick PAA exhibits an exceptional sensitivity of 6.92 kPa^–1^ at a range from 300 Pa to 1.5 kPa. This working range can be interesting to measure small pressure deformation (<1 kPa) and compatible with the monitoring of heartbeat and pulse [296], but, the pressure variation range is small compared with other pressure sensors based on nanomaterials reported in the literature [297]. The structure and the sensing response of the piezoresistive based sensor are presented in Figure 39.

### 4.8. Composite Materials

Motivated by the low costs involved in the PAA/CNFs fabrication process, Kothari et al. [298] studied the potential of the growth of CNF with three different graphitic structures as reinforcement for the PAA/CNFs composite material. MWCNTs were grown immersing the PAA in a Nickel nitrate-based solution and further CVD process was executed. CNFs (with orthogonal graphene structures) were obtained by liquid capillary infiltration liquid crystalline homopolymer of naphthalene through the pores at 300 °C, followed by a carbonization step at 700 °C. Hybrid CNFs-reinforced were obtained firstly by depositing a thin layer of pyrolytic carbon on the pore walls. The two types of CNF-reinforced PAA exhibited similar mechanical properties in all of the tests reported despite the different graphitic orientations. The PAA/MWCNT are more prone to suffer pull-out mechanical failure than the other CNFs reinforcements, but the MWCNTs improves the toughening properties. Even if the CNFs did not provide toughening, the other properties reported by Kothari et al. [298] shed light on the use of nanocarbon structures with different geometries as a reinforcements to substantially improve the properties of PAA coatings. Motivated by the low costs involved in the PAA/CNFs fabrication process, Kothari et al. [299] studied the potential of the growth of CNF with three different graphitic structures as reinforcement for the PAA/CNFs composite material. MWCNTs were grown immersing the PAA in a Nickel nitrate-based solution and further CVD process was executed. CNFs (with orthogonal graphene structures) were obtained by liquid ca-pillary infiltration liquid crystalline homopolymer of naphthalene through the pores at 300 °C, followed by a carbonization step at 700 °C. Hybrid CNFs-reinforced were obtained firstly by depositing a thin layer of pyrolytic carbon on the pore walls. The two types of CNF-reinforced PAA exhibited similar mechanical properties in all the tests reported despite the different graphitic orientations. The PAA/MWCNT are more prone to suffer pull-out mechanical failure than the other CNFs reinforcements, but the MWCNTs improves the toughening properties. Even if the CNFs did not provide toughening, the other properties reported by Kothari et al. [299] shed light on the use of nanocarbon structures with different geometries as a reinforcements to substantially improve the properties of PAA coatings.

To the best of our knowledge, the only applications based on PAA/DLC composites was developed by Aramesh et al. [217] The PAA/DLC composites are evaluated as biocompatible nanoporous electrodes. The authors prove that carbon coating preserves the structural properties of the PAA while the biochemical and physical properties of the membranes are improved. For instance, the chemical stability (1 < pH < 14) is analysed and cortical neurons were found to attach and spread to the nanocarbon coated electrodes, without adding any biomolecules and applying standard tissue culture protocols. Furthermore, the composite showed extreme resistance to vigorous dry and wet chemical attacks. Then, this set of results is very promising to develop PAA/DLC membranes for cell growth, nerve repair and bionic implants in general [300].

## 5. Final Conclusions and Prospects

The present review gives a broad overview of the versatility of PAA templates for the synthesis of a variety of carbon nanostructures. Despite the attractive platform that PAA provides thanks to the capability to control the main geometrical features, the excellent thermal, mechanical, and chemical stability, during the last decade the number of publications was slightly decreasing. This is probably the consequence of the booming of 2D materials that barely needs a template. Herein, the different strategies to develop PAA/Carbon nanostructures assemblies/composites are discussed in-depth in terms of the different synthesis approaches, the influences of the geometrical features, and the physical/chemical aspects. As a result of the excellent PAA templates properties plus the outstanding characteristics of the carbon nanostructures, several applications have been developed during the last decades. This review aims to highlight that many opportunities and challenges are remaining to be tackled on fundamental issues regarding synthesis aspects. 

During recent years, the development of in situ/*operando* characterization techniques have enabled us to gain a deep understanding of nanomaterials synthesis evolution, chemical and structural stability, and environmental influences [79,80,301]. Environmental studies of PAA/Carbon nanostructures composites under in situ/*operando* conditions will give new insights across the material science field. 

Multiple applications require a collective organization in the direction parallel to the substrate, the development of Lateral-PAA templates is an interesting matrix to synthesize 1D materials. However, further effort must be put to get a better understanding of the pore formation and even more in the synthesis of nanostructures such as CNTs, CNFs and nanowires. In addition, highly ordered three-dimensional interconnected nano-architectures can also be explored as host templates for the catalyst-free synthesis of CNTs or CNFs taking as inspiration the work performed by Martin et al. [302]. The implementation of these kinds of geometries can significantly expand the applications based on PAA devices.

Graphene manipulation recently revolutionized the nanoscience domain. However, all the developed applications of PAA/Graphene assemblies entail a transfer process. In this critical review, some guidelines are given to fabricate PAA/graphene devices exploiting a transfer-free process that have never been fabricated. Certainly, this kind of assembly can trigger electronic or sensing applications.

From a sustainability perspective, it is well known that it’s urgent to address critical energy issues such as climate change and energy security. The scope of the present review is not to evaluate the sustainability of the fabrication of carbon synthesis nanostructures inside PAA templates. However, it is of paramount importance to spread the need for more environmentally friendly and clean industrial technologies and processes. From further insight into this topic, the readers can refer to the work performed by Soares Santos et al. [298]. In this review, a few general remarks are provided. Despite the involved mild temperature synthesis and low toxicity substances applied in the aluminium anodization [298], it is necessary to deploy a clean method for the whole fabrication chain of PAA templates [303]. In most of the works dealing with a two-step anodization method, a chromium-based solution is used for the oxide removal. It’s worth mentioning also that there are environmental concerns regarding the processes of producing the aluminium that is used for anodization [304]. Regarding the carbon nanostructures synthesis, more efforts in Bio-derived carbon nanostructures need to be considered as well [305,306].

The emergence of novel materials such as metals sulphides [307,308,309,310] and metal halide perovskites [311] with different geometries (nanodots, NPs, nanorods, nanoflowers) are very interesting to engineering novel hybrid nanomaterials [311,312,313], the PAA/Carbon nanostructure is an attractive platform to develop hybrid nanomaterials in a regular array with specific dimensions. Which is of interest for the development of energy conversion and storage applications [314]. 

Regarding the technology transfer potential of PAA/Carbon nanostructures, further effort must be performed by the scientific community, where the strength/weakness have to be analyzed in each potential application field. Interestingly, Huang et al. [38] pointed out that to increase the flow from the research to commercial products it is critical to improve the existing functionalization methods to fabricate PAA composite materials with enhanced stability and durability. In this review, the different applications listed are discussed of each described developed application based on PAA/Carbon nanostructures. Table 1 summarizes the strengths, disadvantages, and perspectives/opportunities of the used PAA/carbon nanostructure in the above-mentioned applications.

In conclusion, the present reviews demonstrate that the fascinating PAA/carbon nanostructure materials still require abundant efforts, both, regarding the synthesis of carbon nanostructures within the PAA matrix and the development of a wide variety of applications based on PAA/Carbon nanostructures that can pave the way to breakthroughs in the nanotechnology/nanoscience fields.

## Figures and Tables

**Table 1:** Strength and weakness of the used PAA/carbon nanostructure in the above-mentioned applications.

Application	Strength	Weakness	Perspective/Opportunity
Electronic devices	PAA can offer a collective organization and individual electronic units in each nanopore. The dielectric properties of the alumina can be useful to use as an insulator layer.	The synthesis of semi-conductive carbon nanomaterials is very complicated to precisely control.	Few publications were devoted to these kinds of devices the recent years. Transfer-free graphene nanoribbons or graphene nanodots as proposed in this review can revitalize the electronic applications based on PAA/graphene-based assemblies.
Energy conversion and storage devices	The PAA easily tailored structure and the facility to functionalize the carbon nanostructures leads to already efficient energy storage devices without the utilization of precious metals, generally used as electrodes.	Much progress has been achieved in laboratory research but no commercial products were reported.	More complex PAA nano-architectures needs to be explored to improve the energy storage and conversion technologies [315].
Molecular Transport	The structural and geometrical features are easy to control on the development of a template-assisted catalyst-free CVD approach.	Catalytic synthesis of carbon excludes in this kind of application since the PAA needs to be open from both sides [267].	A graphene transfer-free process can be interesting to fabricate PAA/graphene membranes avoiding the defects that can induce the transferring process, such as wrinkles, holes or contaminants [316].
Photonic devices	Carbon nanostructures can be easily functionalized by the electrodeposition process.	To expose the carbon materials to the environment Lateral-PAA are required and carbon nanostructures emerge from the pores. This horizontal geometry is much complex to precisely control.	Fabrication of 3D-PAA architectures can be interesting to increase the exposure of carbon nanostructures within the host PAA matrix [302].
Gas sensors	Easy implementation and cost-competitive price make PAA/Carbon-based gas sensors an attractive platform to develop the applications.	The electrode configuration is crucial for the interaction between the carbon nanostructure and the gas molecule. Also, in a chemiresitor configuration, the PAA walls can absorb gas analytes decreasing the sensor sensitivity and gas desorption.	The chemical stability of PAA/Carbon nanostructures can be attractive to operate under harsh environments [317].
Biosensing and electrochemical sensing	PAA membrane and graphitic structures both have an extensive surface area that creates additional binding sites to immobilize bioreceptor molecules [318].	Bridging between research lab to real-life applications is still an issue [319].	Further functionalization of PAA/Carbon-based structures can be useful to expand the optical measurement of biomarkers [320].
Pressure sensor	High optical transmittance of PAA and graphene are interesting to develop transparent sensors.	CNTs growth inside PAA templates can be difficultly implemented to develop this kind of application.	Flexible pressure sensors can be explored [321].
Composite materials	The PAA coated carbon can increase the mechanical and tribological characteristics [155].	The catalyst-free template approach used to fabricate PAA/Carbon-based composites leads to carbon nanostructures with more crystal lattice defects.	Electroconductive alumina can be an easily up-scalable method [322]. The bio-compatibility needs to be further studied.

## Data Availability

Not applicable.
